# *Actinidia arguta AaMYB4* Confers Cold and Drought Tolerance Through Up-Regulating Antioxidant Capacity Associated with the ROS Scavenging

**DOI:** 10.3390/plants15162426

**Published:** 2026-08-09

**Authors:** Haotian Feng, Jincheng Wang, Qingyu Kang, Wanda Liu, Yu Wang, Xingguo Li, Wenhui Li, Deguo Han

**Affiliations:** 1 Key Laboratory of Biology and Genetic Improvement of Horticultural Crops (Northeast Region), Ministry of Agriculture and Rural Affairs/National-Local Joint Engineering Research Center for Development and Utilization of Small Fruits in Cold Regions, College of Horticulture, Northeast Agricultural University, Harbin 150030, China; 15776805163@163.com (H.F.); 15645071537@163.com (J.W.); kangqingyu20050419@163.com (Q.K.); xingguoli@neau.edu.cn (X.L.); 2 Horticulture Branch, Heilongjiang Academy of Agricultural Sciences, Harbin 150069, China; liuwanda@haas.cn (W.L.); haaslwd@126.com (Y.W.)

**Keywords:** *Actinidia arguta*, *AaMYB4*, ROS, stress tolerance, ABA

## Abstract

*Actinidia arguta* possesses great commercial value as an economically important fruit crop, which accumulates abundant nutrients and bioactive components with medicinal potential. However, adverse abiotic environments, especially cold and drought stress, severely restrict its vegetative growth, reproductive development and fruit yield. Numerous studies have established MYB transcription factors as core regulators of plant abiotic stress adaptation. Here, we cloned *AaMYB4* from *A. arguta* ‘Fenglü’ and systematically characterized its function in cold and drought tolerance. *AaMYB4* encodes a 241-amino-acid R2R3-MYB protein localized to the nucleus, with highest expression in stems and young leaves. Its transcription is markedly induced by cold, drought and abscisic acid (ABA) within 24 h with a single peak expression pattern. Heterologous overexpression of *AaMYB4* in *Arabidopsis* alleviated cold-induced oxidative damage, accompanied by reduced malondialdehyde (MDA) and reactive oxygen species (ROS) accumulation as well as increased proline contents and enhanced superoxide dismutase (SOD), peroxidase (POD), and catalase (CAT) activities. Virus-induced gene silencing (VIGS)-mediated silencing of *AaMYB4* impaired cold tolerance in *Actinidia arguta* seedlings, while stable *AaMYB4* overexpression significantly improved plant survival and physiological performance under cold and drought conditions, concurrent with attenuated ROS accumulation. At the transcriptional level, *AaMYB4* overexpression is positively associated with elevated transcript levels of stress marker genes in the ABA signaling and ICE1-CBF-COR pathways. This study lays a theoretical foundation for exploring abiotic stress tolerance mechanisms and conducting stress-resistant molecular breeding in *A. arguta*.

## 1. Introduction

Plants are continuously exposed to a wide range of environmental stimuli. Biotic and abiotic stresses are major constraints on crop growth, yield, and quality in agricultural production. Biotic stresses include pathogen infection, insect herbivory, and parasitic plant infestation, which cause disease, stunt plant growth, and in severe cases lead to plant death [1,2,3,4,5]. In contrast, abiotic stresses stem from non-living environmental factors, including water deficit, extreme temperature, and osmotic imbalance. Upon exposure to stress, plants rapidly perceive external signals, activate intracellular signaling cascades, and trigger a suite of physiological, biochemical, and molecular adaptive responses [6,7,8].

Low temperature is one of the most prevalent and detrimental abiotic stresses, representing a major constraint on the geographical distribution, growth periodicity, and yield of perennial horticultural crops in temperate zones [9,10]. Plant cold tolerance refers to the ability to adapt to low temperatures via morphological adjustments and reprogramming of physiological and biochemical metabolism [11]. Low-temperature signals induce both conserved and specific physiological responses at the cellular, tissue, and organ levels, including the accumulation of osmoprotectants, activation of antioxidant enzymes, and stabilization of membrane systems [12,13,14,15]. At the molecular level, plant cold adaptation is mainly mediated by CBF-dependent and CBF-independent signaling pathways. In the canonical CBF pathway, cold signals induce the expression of CBF transcription factors, which bind to C-repeat (CRT)/dehydration-responsive element (DRE) cis-elements in the promoters of downstream COR genes to activate cold-responsive gene expression and enhance cellular freezing tolerance. The CBF-independent regulatory network involves calcium signaling, the abscisic acid (ABA) cascade, and mitogen-activated protein kinase (MAPK) pathways, which collectively fine-tune cold stress responses in plants [16,17,18].

Drought is another pervasive abiotic stress, caused by insufficient soil moisture, excessive transpiration, or impaired water homeostasis, which results in severe cellular water deficit in plant tissues. At the molecular level, plant drought responses rely on signal perception, signal transduction, and the regulation of downstream stress-responsive genes, mediated primarily by ABA-dependent and ABA-independent pathways that together form a complex regulatory network for drought tolerance. In the ABA-dependent pathway, drought stress strongly triggers endogenous ABA biosynthesis and accumulation. ABA binds to specific intracellular receptors to activate downstream phosphorylation cascades, thereby regulating the expression of core transcription factor families, including bZIP, MYB, NAC, WRKY, and NF-Y, to mount adaptive responses to drought [19,20]. In the ABA-independent pathway, DRE/CRT cis-acting elements and AP2/ERF-type DREB/CBF transcription factors serve as central regulators [21,22].

A typical MYB protein contains three core functional regions: a highly conserved N-terminal DNA-binding domain (DBD), a central transcriptional activation domain, and a C-terminal negative regulatory domain [23,24]. The MYB DBD comprises 1 to 4 conserved R repeats, each approximately 51–53 amino acids in length. Based on the number of R domains, the plant MYB family is classified into four subfamilies: 1R-MYB, R2R3-MYB, R1R2R3-MYB, and 4R-MYB [25,26,27]. Accumulating evidence demonstrates that MYB transcription factors act as critical regulatory hubs in plant stress tolerance [28]. By binding to specific cis-elements in the promoters of downstream functional genes, MYB proteins modulate target gene transcription, thereby regulating osmotic adjustment, antioxidant defense, phytohormone signal transduction, and stress adaptation. In the plant cold response, MYB family members participate in both CBF-dependent and CBF-independent pathways, mediating osmoprotectant biosynthesis, reactive oxygen species (ROS) scavenging, and membrane stability maintenance. In *Arabidopsis thaliana*, *AtMYB96* acts as a positive regulator of cold tolerance: it enhances plant cold tolerance by activating CBF pathway genes and promoting the accumulation of osmoprotectants such as proline and glycine betaine, while simultaneously coordinating ABA signal transduction to fine-tune cold stress adaptation [29].

With respect to drought stress regulation, MYB transcription factors broadly mediate both ABA-dependent and ABA-independent signaling pathways to modulate plant water homeostasis, osmotic balance, and antioxidant capacity, thereby conferring enhanced drought tolerance. In the model plant *Arabidopsis thaliana*, several MYB genes have been characterized as positive regulators of drought tolerance, including *AtMYB44*, *AtMYB74*, and *AtMYB102* [30]. *AtMYB60* and *AtMYB96* fine-tune drought responses by integrating ABA and auxin signaling pathways to control stomatal movement and transpirational water loss [31]. Moreover, *AtMYB41* shows negligible expression under normal growth conditions, but its transcription is strongly induced by drought, ABA, and salt stress, indicative of its critical role in plant abiotic stress adaptation [32,33]. In crop species, cotton *GhMYB6* enhances drought tolerance by regulating the expression of stomatal regulatory genes, reducing foliar water transpiration, and mitigating drought-induced growth inhibition [34]. Taken together, MYB transcription factors have evolutionarily conserved functions in regulating drought stress responses across diverse plant taxa.

*Actinidia arguta* (commonly known as kiwiberry) is an economically important perennial fruit crop with favorable horticultural traits, including robust tolerance to biotic and abiotic stresses such as pathogens, drought, cold, and saline-alkali conditions [35,36,37,38]. Existing studies on kiwifruit MYB transcription factors have focused primarily on *Actinidia chinensis*. Regarding cold stress responses, the R2R3-MYB gene *AcMYB78* can directly bind to the promoters of AcCBF1/2 and AcRAP2.1, negatively regulating freezing tolerance in kiwifruit by repressing *AcRAP2.1* and activating AcCBFs, representing the first characterized MYB-mediated regulatory mechanism of the CBF pathway in kiwifruit cold responses [39]. In the context of drought adaptation, *PdbMYB6* improves drought tolerance by regulating ROS scavenging, osmotic balance, and stomatal aperture [40]. Nevertheless, research on MYB-mediated stress regulation in *A. arguta* remains very limited, and is restricted to preliminary phenotypic observations under cold stress. The core regulatory functions, downstream target genes, and associated signaling pathways of *A. arguta* MYB members under cold and drought stresses remain largely uncharacterized.

In this study, we isolated and cloned a stress-responsive R2R3-MYB gene, *AaMYB4*, from *A. arguta*. We systematically characterized its subcellular localization, sequence features, and tissue-specific and stress-induced expression patterns. We further investigated its function in cold and drought tolerance via heterologous expression in Arabidopsis and physiological assays, and explored its association with the ABA and ICE1-CBF-COR signaling pathways. This work aims to elucidate the regulatory role of *AaMYB4* in abiotic stress adaptation in *A. arguta*, and provide a theoretical basis for dissecting stress tolerance mechanisms and conducting stress-resistant molecular breeding of kiwiberry.

## 2. Results

### 2.1. Bioinformatics Analysis and Cloning of the AaMYB4 Gene

The full-length coding sequence of *AaMYB4* was isolated from *Actinidia arguta* cultivar ‘Fenglü’. The resulting nucleotide sequence was translated into its corresponding amino acid sequence using DNAMAN 5.2 software (Figure S1). The complete coding region of *AaMYB4* is 726 bp in length.

**Figure 1:**
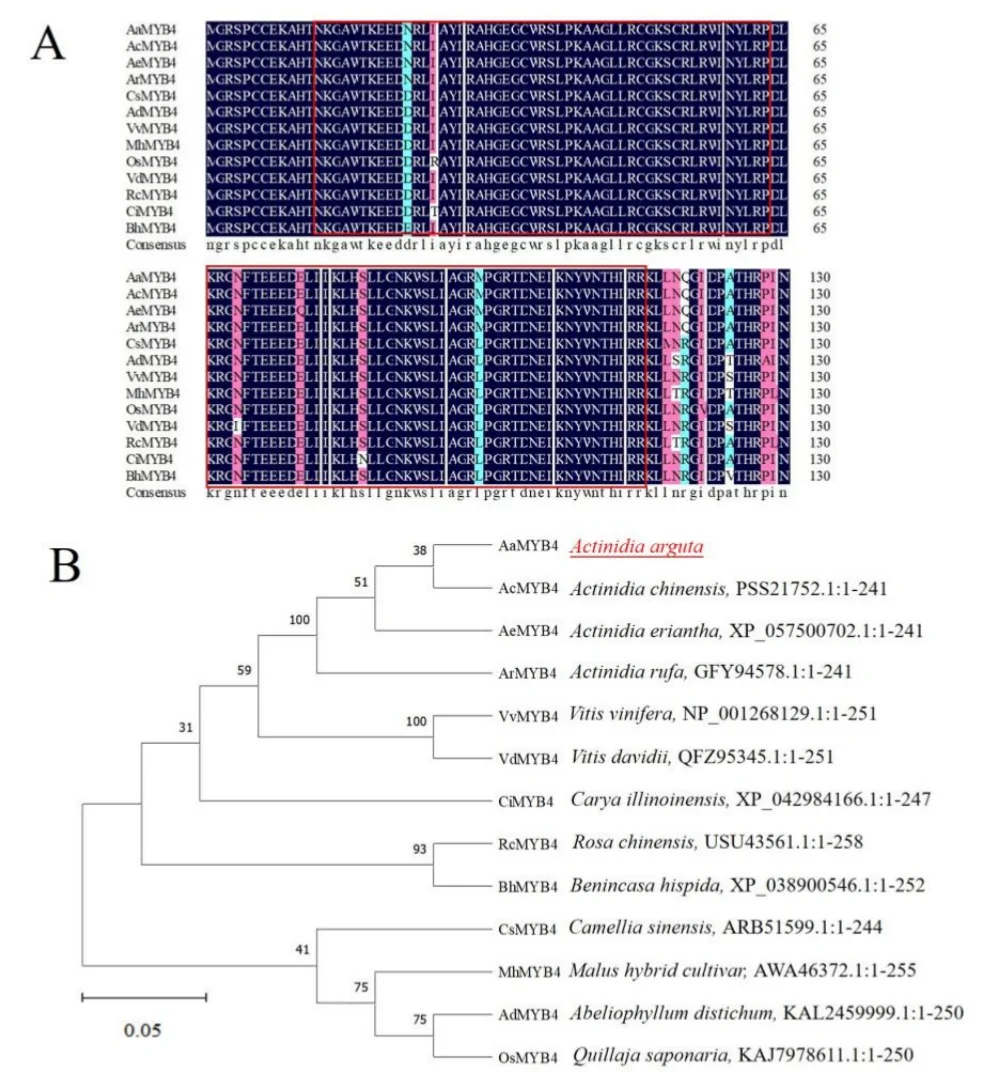
Multiple sequence alignment and phylogenetic analysis of AaMYB4 proteins from various plant species. (**A**) Amino acid sequence alignment of AaMYB4 and homologous MYB4 proteins from different plant species. The red boxes mark the two highly conserved MYB DNA-binding domains. (**B**) Phylogenetic tree constructed with AaMYB4 and MYB4 homologous proteins from diverse plant species. The gene from *Actinidia arguta* is highlighted in red. The bootstrap values are displayed on each branch. MEGA 7.0 software was used to construct the phylogenetic tree, and the scale bar indicates the genetic distance.

Analysis via the ExPASy ProtParam tool revealed that the deduced AaMYB4 protein comprises 241 amino acid residues and harbors two conserved SANT motifs (Figure 1A). Further physicochemical characterization with ExPASy tools indicated a theoretical molecular weight of 26,999.69 Da for AaMYB4, with an isoelectric point (pI) of 8.97.

In terms of amino acid composition, leucine (Leu, 9.5%), glycine (Gly, 9.1%), lysine (Lys, 7.9%) and arginine (Arg, 7.5%) represent the most abundant residues. The protein has a grand average of hydropathicity (GRAVY) value of −0.720 and an instability index of 46.77.

Homology searches for the AaMYB4 protein sequence were conducted using the BLASTp 2.14.0 tool hosted on the National Center for Biotechnology Information (NCBI) platform (https://blast.ncbi.nlm.nih.gov/Blast.cgi (accessed on 10 May 2026)). High sequence similarity between AaMYB4 from *A. arguta* and MYB4 homologs across diverse plant taxa, including AcMYB4 from *Actinidia chinensis*, ArMYB4 from *Actinidia rufa*, AcMYB4 from *Actinidia eriantha*, VvMYB4 from *Vitis vinifera*, VdMYB4 from *Vitis davidii*, CiMYB4 from *Carya illinoinensis*, RcMYB4 from *Rosa chinensis*, BhMYB4 from *Benincasa hispida*, CsMYB4 from *Camellia sinensis*, MhMYB4 from a *Malus hybrid* cultivar, AdMYB4 from *Abeliophyllum distichum*, and QsMYB4 from *Quillaja saponaria*.

All these homologous proteins contain two conserved SANT domains, spanning approximately amino acid positions 13 to 63 and 66 to 114. Multiple sequence alignment was performed using MEGA 11.0 software, based on which a phylogenetic tree was constructed (Figure 1B).

**Figure 2:**
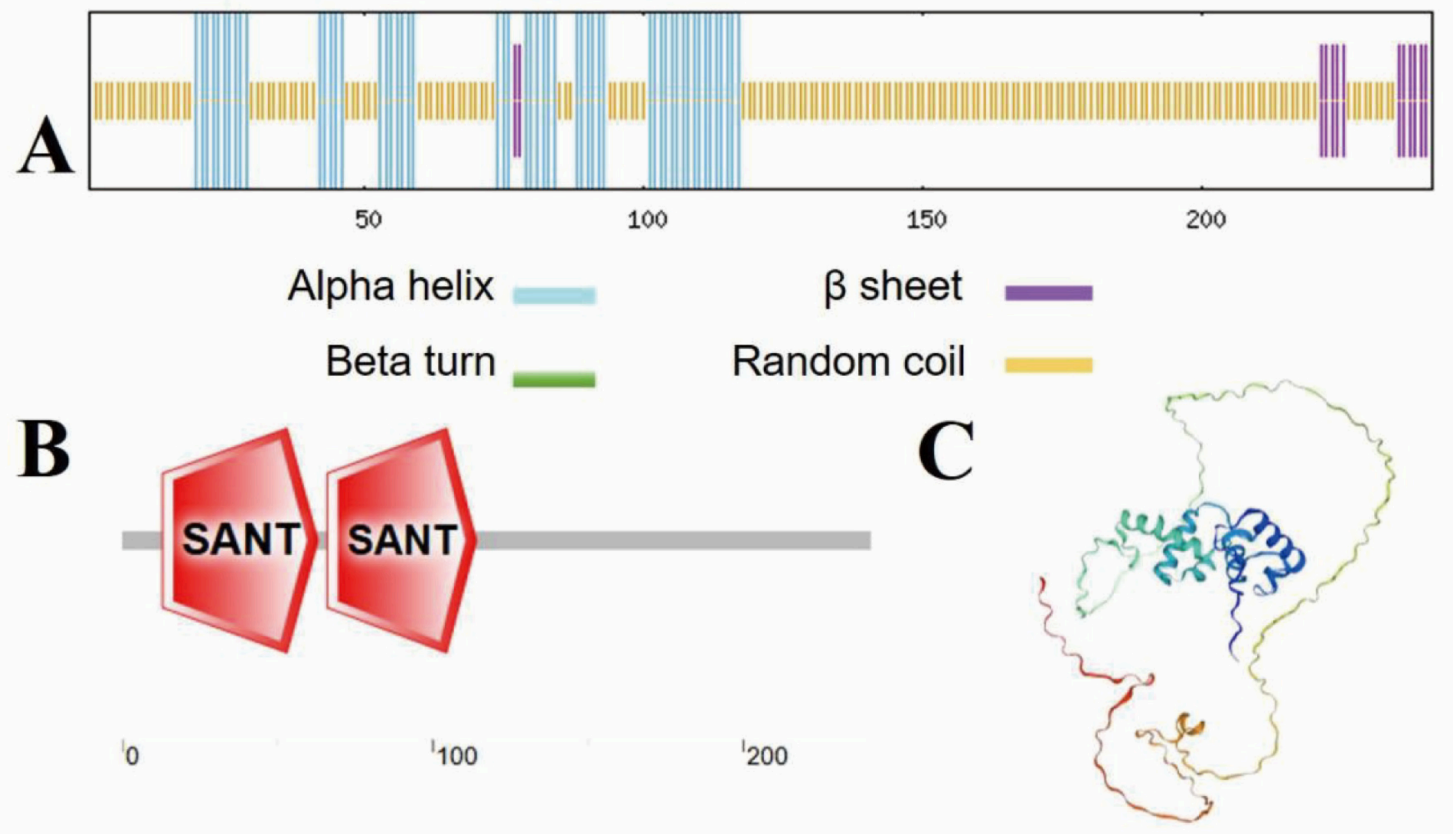
Prediction of secondary structure as well as tertiary structure of AaMYB4 protein. (**A**) Prediction of the secondary structure of AaMYB4. Alpha helices are marked in light blue, β-sheets in purple, beta turns in green, and random coils in yellow. (**B**) Conserved domain analysis of the AaMYB4 protein. (**C**) Homology modeling of the three-dimensional tertiary structure of AaMYB4 protein.

The secondary structural elements of the AaMYB4 protein were predicted using the online SOPMA tool (https://npsa-prabi.ibcp.fr/ (accessed on)). As shown in Figure 2A, the relative proportions of the different structural components are as follows: random coils (73.44%), followed by α-helices (23.65%), and extended strands (2.90%).

On the basis of its secondary structure, the polypeptide chain further folds and assembles to form a native three-dimensional tertiary conformation. Conserved domain analysis with the SMART tool confirmed that AaMYB4 harbors two SANT domains spanning amino acid residues 13 to 63 and 66 to 114 (Figure 2B).

The tertiary structure of AaMYB4 was homology-modeled using the online SWISS-MODEL platform (Figure 2C).

### 2.2. Subcellular Localization of the AaMYB4 Protein

To determine the subcellular localization of the AaMYB4 protein, we generated the transient fusion expression vector pCAMBIA1300-AaMYB4::GFP, with the empty 35S::GFP vector serving as the negative control.

Both the 35S::AaMYB4-GFP fusion plasmid and empty 35S::GFP control vector were transformed into *Agrobacterium tumefaciens*. Subsequently, the resulting bacterial suspensions were infiltrated into the abaxial epidermal cells of Nicotiana benthamiana leaves for subcellular localization analysis.

**Figure 3:**
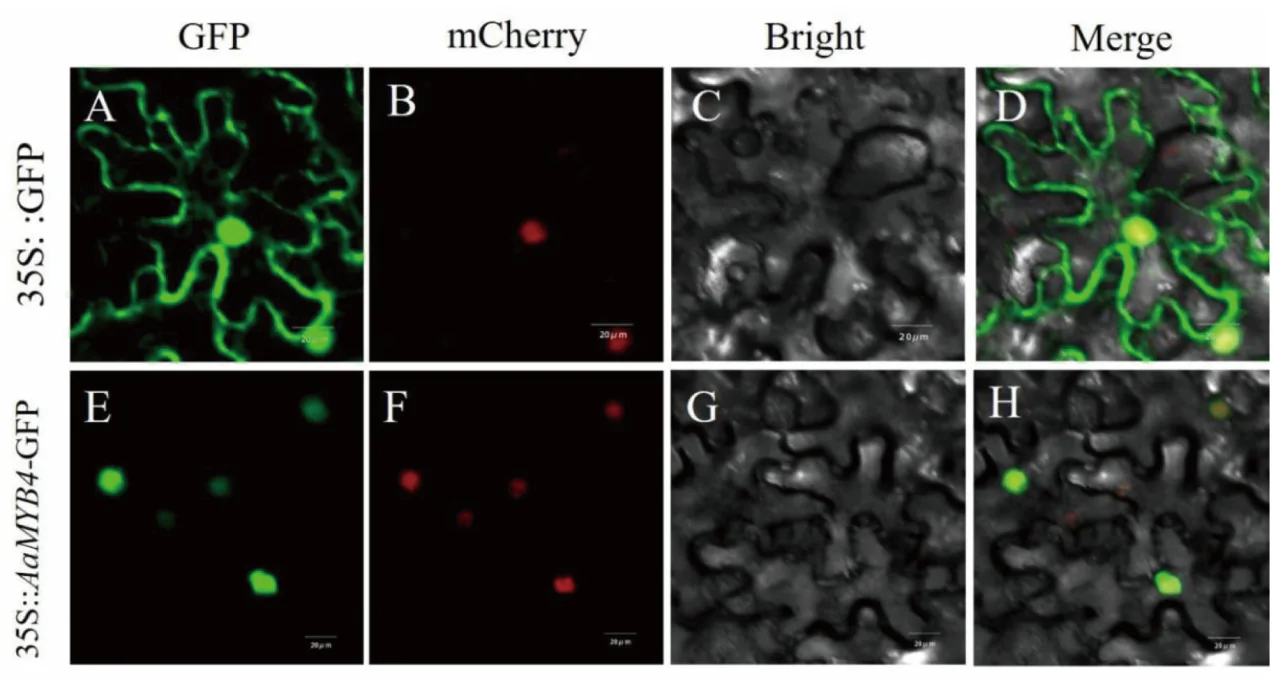
Subcellular localization of AaMYB4 protein in tobacco epidermal cells. The 35S::*AaMYB4*-GFP fusion vector and empty 35S::GFP control were transiently expressed in tobacco leaves. (**A**,**E**) GFP fluorescence; (**B**,**F**) mCherry nuclear marker fluorescence; (**C**,**G**) bright field; (**D**,**H**) merged images. Yellow fluorescence indicates the co-localization of GFP and mCherry signals. Scale bars = 20 µm.

Laser confocal microscopy analysis revealed that in control leaves expressing 35S::GFP, GFP fluorescence was detected in both the plasma membrane and the nucleus simultaneously. In contrast, the GFP signal derived from pCAMBIA1300-AaMYB4::GFP was exclusively localized to the nucleus, and co-localized with the red fluorescence of the nuclear marker mCherry, producing yellow signals in the merged channel (Figure 3).

### 2.3. Tissue-Specific Expression Analysis of the AaMYB4 Gene

**Figure 4:**
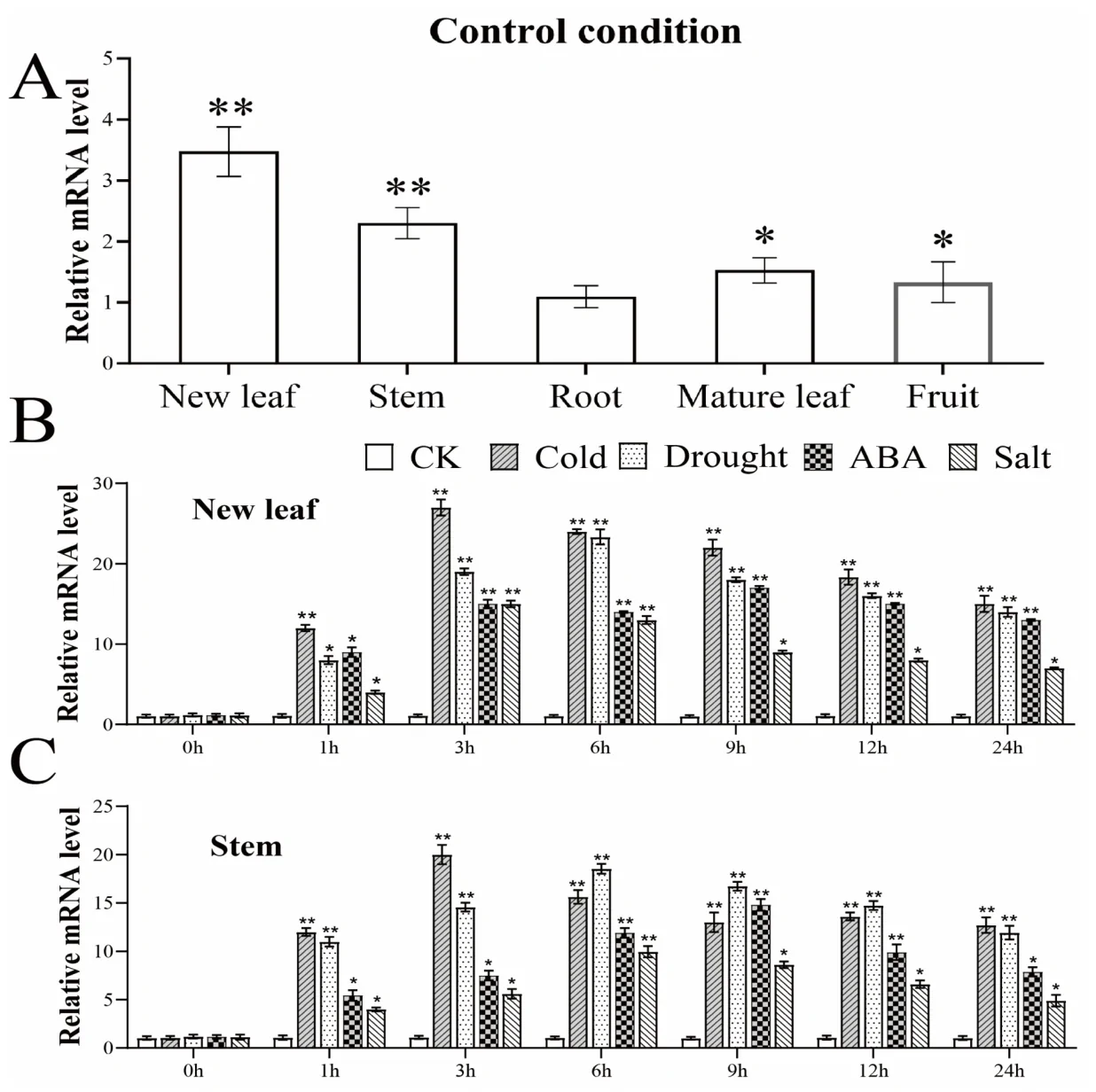
Tissue and stress-responsive expression of *AaMYB4* in *Actinidia arguta*. (**A**) Transcript levels of *AaMYB4* in roots, stems, young/mature leaves and fruits. (**B**,**C**) Time-series expression of *AaMYB4* in young leaves (**B**) and stems (**C**) under 4 °C cold, 6% polyethylene glycol (PEG) drought, 100 μM ABA and 200 mM NaCl salt treatments. Data represent mean ± standard error (SE) from three independent biological replicates. Asterisks indicate significant differences relative to the control (root in (**A**); 0 h samples in (**B**,**C**)). * *p* ≤ 0.05, ** *p* ≤ 0.01 (Student’s *t*-test).

Quantitative real-time PCR (qRT-PCR) was performed to quantify the transcript abundance of *AaMYB4* across different tissues of ‘Fenglü’ kiwiberry seedlings, including young leaves, stems, roots, mature leaves, and fruits (Figure 4A).

‘Fenglü’ kiwiberry seedlings were subjected to four abiotic stress treatments: low temperature (4 °C), drought (6% PEG6000), exogenous (ABA, 100 μM), and high salinity (200 mM NaCl). Samples were collected at 0, 1, 3, 6, 9, 12, and 24 h after treatment initiation. Total RNA was extracted from the harvested samples and reverse-transcribed into cDNA for subsequent qRT-PCR analysis.

*AaMYB4* expression was induced by all four stress treatments. Over the 24-h treatment period, *AaMYB4* expression exhibited a unimodal pattern in both young leaves and stems, with peak timing consistent between the two tissues. As shown in Figure 4B,C, *AaMYB4* displayed stronger responsiveness to cold and drought stresses. Upon exposure to the four stress treatments, *AaMYB4* transcript levels in young leaves showed a pattern of initial increase followed by decline. Specifically, expression peaked at 3 h under cold stress, 6 h under drought stress, 9 h under ABA treatment, and 3 h under salt stress. In stem tissues, the corresponding expression peaks occurred at 3 h (cold), 6 h (drought), 9 h (ABA), and 6 h (salt). After 3 h of 4 °C cold treatment, *AaMYB4* transcript levels in young leaves reached their maximum, which was 7.714-fold higher than that in untreated controls; the peak expression level in stems was 6.826-fold higher than the baseline control level (Figure 4B,C).

### 2.4. Instantaneous Transformation of AaMYB4 in Actinidia arguta Leaves for Responding to Low-Temperature Stress

To investigate the role of *AaMYB4* in low-temperature stress responses, we performed transient transformation assays using detached leaves of *Actinidia arguta*.

The full-length coding sequence (CDS) of *AaMYB4* was inserted into the overexpression vector pCAMBIA1300 to generate the recombinant overexpression construct pCAMBIA1300-*AaMYB4*, with the empty pCAMBIA1300 vector serving as the empty vector control. Agrobacterium-mediated vacuum infiltration was used to deliver the constructs into detached leaves of *A. arguta* ‘Fenglü’, which were then incubated in a light incubator for 3 d to maintain viability (Figure S4A) prior to 4 °C cold treatment. Leaf samples were collected at 4 h post-treatment to determine the relative expression level of *AaMYB4*. Two independent transient overexpression groups (MYB4-OE1 and MYB4-OE2) were generated (Figure S4C).

**Figure 5:**
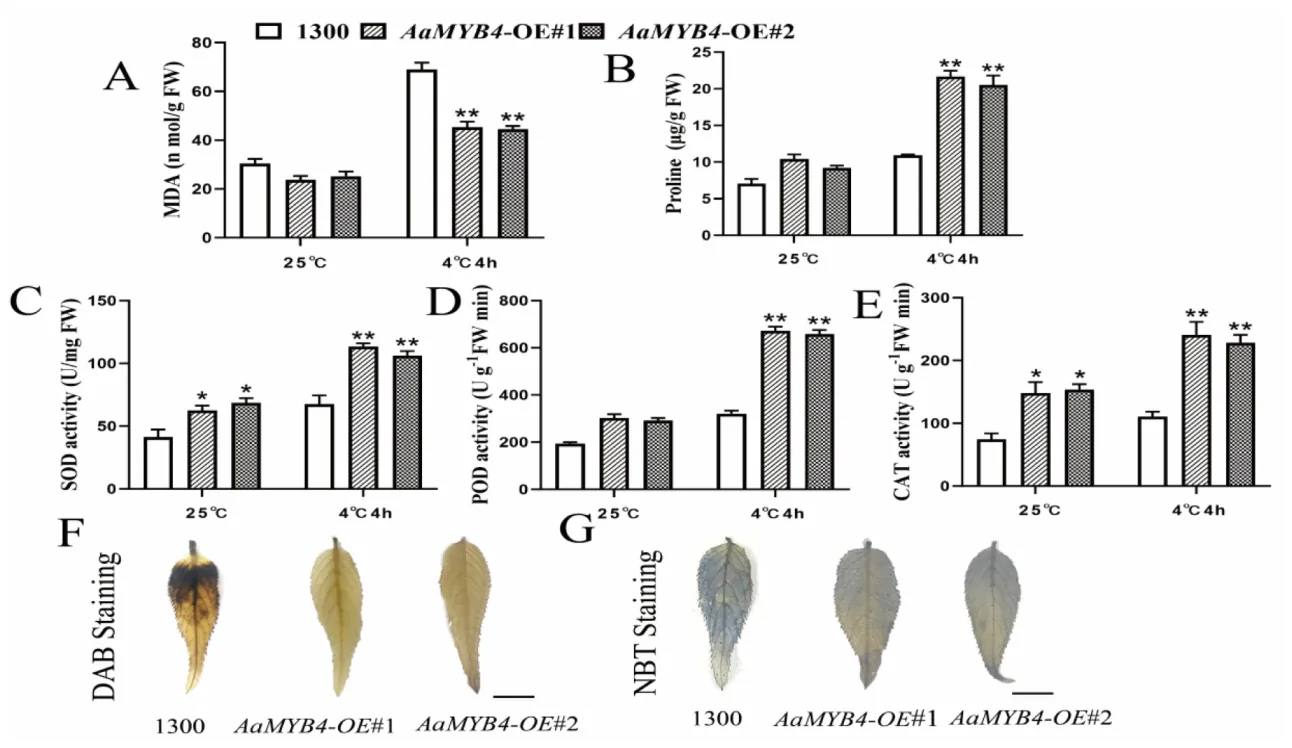
Overexpression of *AaMYB4* alters leaf physiological parameters and ROS levels. (**A**,**B**) MDA and proline contents in leaf tissues. (**C**–**E**) Activities of SOD, POD and CAT antioxidant enzymes. (**F**,**G**) DAB and NBT staining for ROS accumulation. Data represent mean ± standard error (SE) from three independent biological replicates. Asterisks indicate significant differences compared with the empty vector control (1300). * *p* ≤ 0.05, ** *p* ≤ 0.01 (Student’s *t*-test).Scale bars represent 1 cm.

**Figure 6:**
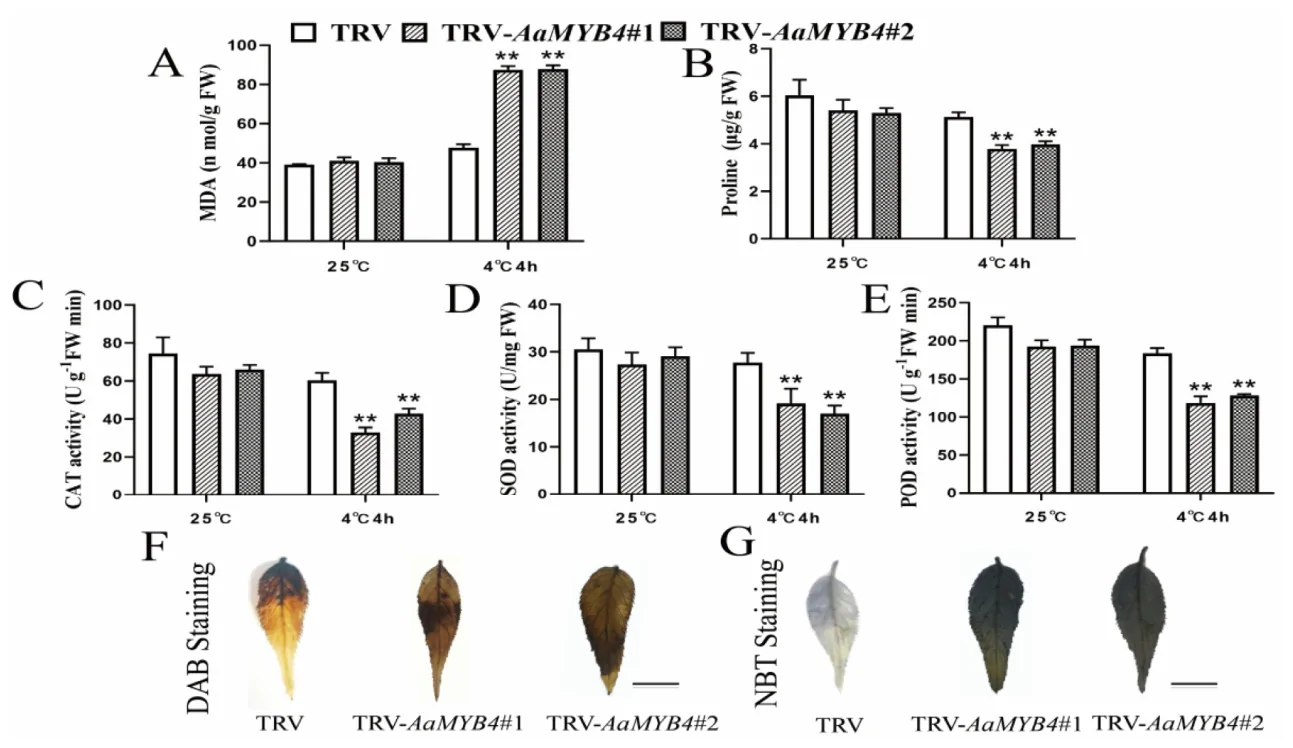
Silencing *AaMYB4* alters leaf physiological parameters and ROS levels. (**A**,**B**) MDA and proline contents in leaf tissues. (**C**–**E**) Enzymatic activities of SOD, POD and CAT. (**F**,**G**) DAB and NBT histochemical staining for ROS accumulation. Data represent mean ± standard error (SE) from three independent biological replicates. Asterisks indicate significant differences between each silenced line and the TRV control within the identical temperature treatment. ** *p* ≤ 0.01 (Student’s *t*-test).Scale bars represent 1 cm.

After cold treatment, MYB4-OE leaves exhibited significantly lower malondialdehyde (MDA) content (Figure 5A), along with higher proline content and enhanced activities of three core antioxidant enzymes: superoxide dismutase (SOD), peroxidase (POD), and catalase (CAT), compared with control leaves (Figure 5C–E). Histochemical staining with 3,3′-diaminobenzidine (DAB) and nitro blue tetrazolium (NBT) revealed fewer brown and blue precipitates in MYB4-OE leaves, indicating reduced accumulation of ROS including H_2_O_2_ and O_2_^−^ (Figure 5F,G). For virus-induced gene silencing (VIGS) assays, a specific antisense fragment targeting the 5′-UTR of *AaMYB4* was inserted into the pTRV2 vector to generate the recombinant silencing construct pTRV2-*AaMYB4*. Using the empty pTRV2 vector as a control, *A. arguta* ‘Fenglü’ seedlings were infiltrated via Agrobacterium-mediated vacuum infiltration, rinsed with distilled water, transferred to solid growth medium, and cultured in the dark for 3 d. Following incubation, seedlings were exposed to 4 °C cold stress, and the relative expression of *AaMYB4* was quantified at 4 h after treatment. Two independent silencing groups (MYB4-TRV1 and MYB4-TRV2) were obtained; *AaMYB4* transcript levels in MYB4-TRV leaves were significantly lower than those in the pTRV2 control (TRV) (Figure S4D), and no obvious phenotypic differences were observed between groups at 25 °C. After 4 °C treatment, MYB4-TRV seedlings exhibited severe leaf wilting and necrosis, whereas control seedlings showed only slight water-soaked symptoms and maintained normal growth (Figure S4B). MYB4-TRV leaves had significantly higher MDA content (Figure 6A) and markedly lower proline content as well as reduced SOD, POD, and CAT activities compared with TRV control leaves (Figure 6C–E). Consistently, DAB and NBT staining showed more intense brown and blue precipitates in MYB4-TRV leaves (Figure 6F,G).

### 2.5. Overexpression of AaMYB4 Enhances Cold and Drought Stress Tolerance in Arabidopsis thaliana

To investigate the effects of *AaMYB4* overexpression on cold and drought tolerance in *Arabidopsis thaliana*, we generated the recombinant overexpression vector *AaMYB4*-pCAMBIA1300. Both the empty pCAMBIA1300 vector and the recombinant AaMYB4-pCAMBIA1300 construct were separately transformed into wild-type (WT) *A. thaliana* to generate transgenic lines. T1 transgenic seedlings and empty vector control lines (UL) were subjected to kanamycin resistance screening to identify positive transformants harboring the *AaMYB4* overexpression cassette.

Total RNA was extracted from positive seedlings, and the transcript levels of the target gene were determined by qRT-PCR. WT plants and UL lines were included as negative controls, both of which showed no detectable *AaMYB4* expression. In contrast, T2 positive transgenic lines exhibited varying *AaMYB4* transcript levels. To confirm stable genomic integration of the transgene, genomic DNA was isolated from leaf tissues of eight putative *A. thaliana* transgenic lines (S1–S8), and molecular validation was performed via polymerase chain reaction (PCR) using the *AaMYB4*-F/R primer pair (Table S1). Gel electrophoresis analysis confirmed that the expected target bands were amplified in all eight transgenic lines, whereas no corresponding amplicons were detected in WT and UL control plants (Figure S5D). Subsequent transcript analysis revealed that three lines (S1, S3, and S5) exhibited significantly higher *AaMYB4* expression than the other transgenic lines (Figure S5C). Accordingly, these three high-expression lines were selected for successive propagation to generate homozygous T3 lines with stable inheritance, which were used for subsequent functional assays (Figure S5B). Subsequently, homozygous T3 transgenic lines (S1, S3, and S5), along with WT and UL controls of uniform growth status, were used for phenotypic assays of cold and drought stress tolerance.

**Figure 7:**
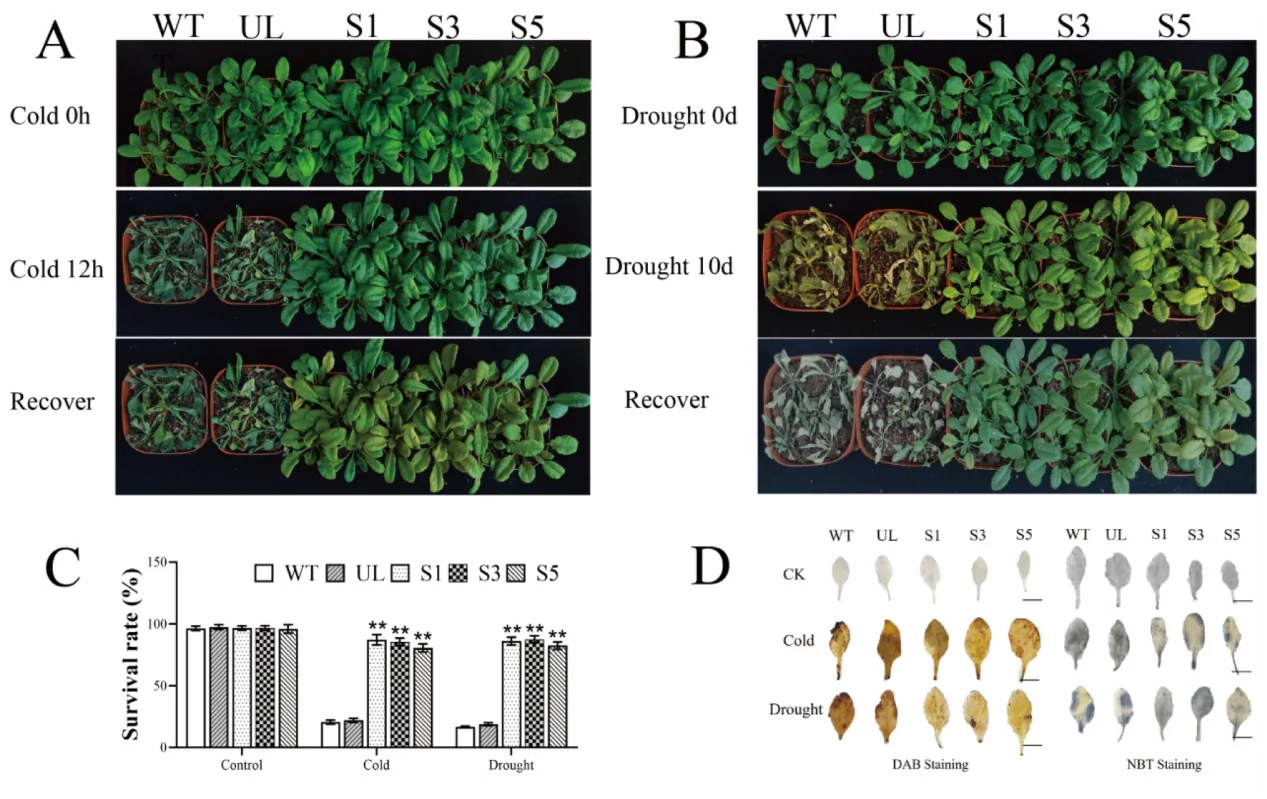
Phenotypes, survival rates and ROS accumulation of WT, UL and *AaMYB4*-silenced lines (S1/S3/S5) under cold and drought stress. (**A**,**B**) Plant morphology before stress, after 12 h cold/10 d drought and recovery; (**C**) survival statistics; (**D**) DAB and NBT staining for H_2_O_2_ and O_2_^−^. Data represent mean ± standard error (SE) from three independent biological replicates. Asterisks indicate significant differences compared with the WT control. ** *p* ≤ 0.01 (Student’s *t*-test). Images in (**A**–**C**) are representative results from three independent experiments.Scale bars represent 1 cm.

Uniformly growing plants from each line were assigned to two parallel experimental groups (Figure 7). One group was exposed to 4 °C cold stress for 12 h, and the other was subjected to drought stress by continuous water withholding for 10 d. Phenotypic changes were monitored and recorded at three key stages: before stress treatment, immediately after stress cessation, and after a 7-day recovery under normal growth conditions. The survival rate of each line was statistically calculated after the 7-day recovery period.

Under normal growth conditions, WT, UL and transgenic lines showed no visible phenotypic differences and comparable growth status. Following exposure to cold and drought stresses, all lines showed varying degrees of leaf damage, including wilting and chlorosis, but the severity of damage differed significantly between the transgenic lines and the control groups. Whereas WT and UL seedlings developed severely wilted and chlorotic leaves, *AaMYB4*-overexpressing lines retained relatively green and healthy foliage (Figure 7A,B). After the low-temperature treatment followed by a 7-day recovery period, the majority of transgenic seedlings maintained vigorous growth. The survival rates of lines S1, S3, and S5 reached 90.56%, 87.64%, and 86.72%, respectively. In contrast, WT and UL seedlings exhibited much lower survival rates of only 22.14% and 25.42%, respectively. Likewise, after 10 d of drought stress followed by a 7-day recovery with rewatering, the average survival rates of the WT and UL groups were only 19.79% and 21.37%, whereas the transgenic lines displayed strong recovery capacity, with survival rates of 89.96%, 88.38%, and 87.12% for S1, S3, and S5, respectively (Figure 7C).

Histochemical staining was performed to visualize in situ accumulation of ROS in *A. thaliana* leaves. Leaves from control, cold-treated, and drought-treated groups were stained with DAB and NBT to detect the accumulation of hydrogen peroxide (H_2_O_2_) and superoxide anion (O_2_^−^), respectively (Figure 7D). Under non-stress conditions, leaves from all lines showed negligible staining, indicating comparably low basal ROS levels across WT, UL, and AaMYB4-overexpressing lines (S1, S3, S5). Following exposure to 4 °C cold stress for 12 h or 10 d of drought, obvious stained precipitates were observed in leaves of all genotypes.

**Figure 8:**
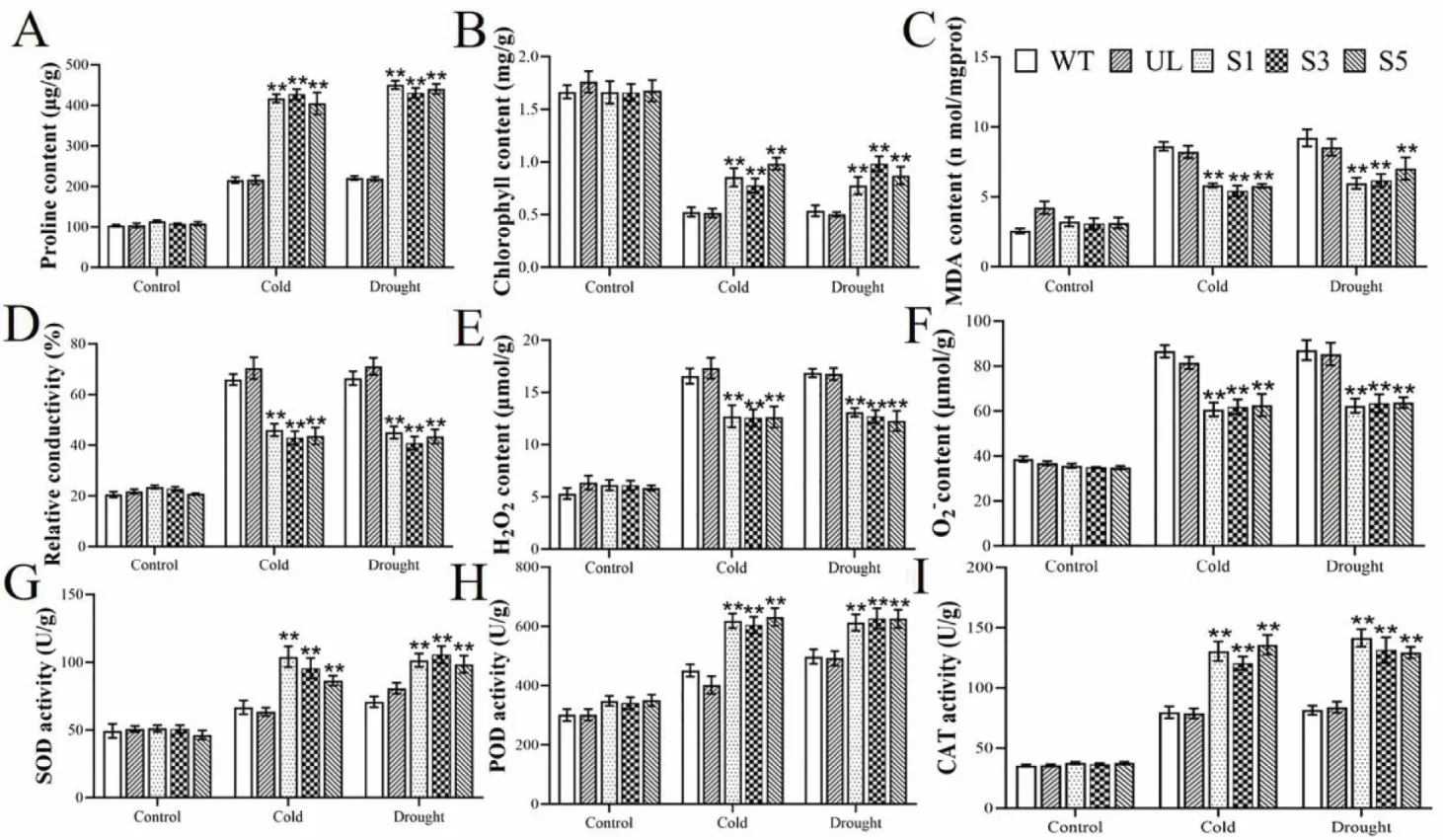
Physiological indices regulated by *AaMYB4* in cold- and drought-stressed *Arabidopsis*. (**A**–**I**) Contents of proline, chlorophyll, MDA, electrolyte leakage, H_2_O_2_, O_2_^−^, and activities of SOD, POD, CAT in WT, UL and three *AaMYB4* overexpression lines (S1/S3/S5). Data represent mean ± SE from three independent biological replicates. Asterisks denote significant differences relative to WT under the same treatment. ** *p* ≤ 0.01 (Student’s *t*-test).

However, staining intensity varied significantly among groups. Leaves of the three *AaMYB4*-overexpressing lines showed much weaker brown and blue staining than WT and UL controls, indicating significantly reduced H_2_O_2_ and O_2_^−^ accumulation in transgenic leaves under stress conditions. Under normal growth conditions, WT, UL, and *AaMYB4*-overexpressing lines showed comparable physiological status, with no significant differences in chlorophyll and proline contents, or in the activities of SOD, POD, and CAT. In sharp contrast, cold and drought stresses induced pronounced physiological changes across all tested genotypes. Under stress, *AaMYB4*-overexpressing lines accumulated more proline and chlorophyll, showed higher SOD, POD and CAT activities, and had lower MDA, H_2_O_2_ and O_2_^−^ levels than controls (Figure 8A,B,G–I). For stress damage-related indicators, transgenic lines exhibited lower MDA, H_2_O_2_, and O_2_^−^ levels relative to the controls.

### 2.6. AaMYB4 Is Associated with Altered Expression of Cold- and Drought-Tolerance-Related Genes in Arabidopsis

**Figure 9:**
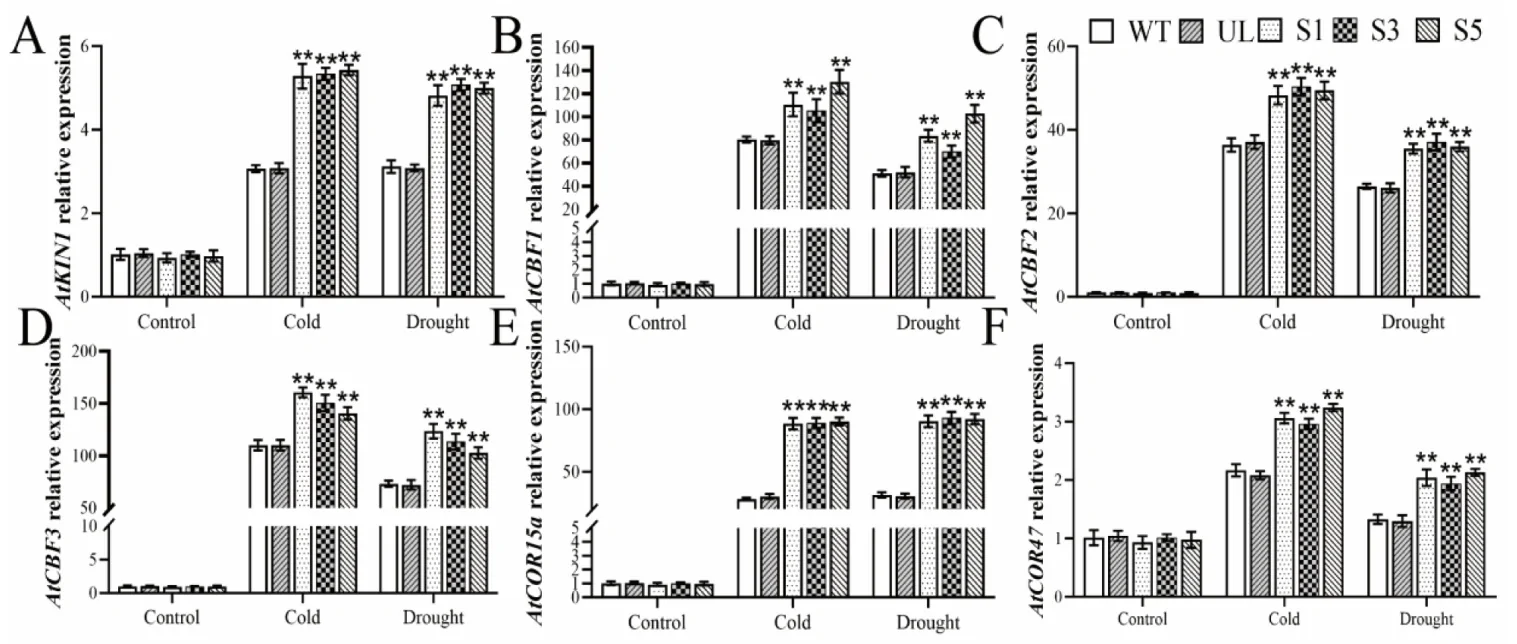
*AaMYB4*-mediated expression of cold/drought marker genes. (**A**–**F**) Relative expression of six stress genes in WT, UL and transgenic plants under control, cold and drought treatments. Data represent mean ± standard error (SE) from three independent biological replicates. Asterisks indicate significant differences compared with the WT group within the identical treatment condition. ** *p* ≤ 0.01 (Student’s *t*-test).

To further elucidate the molecular mechanism underlying *AaMYB4*-mediated cold and drought tolerance, we analyzed the expression profiles of key downstream stress-responsive marker genes in transgenic *A. thaliana*. Six well-characterized stress-responsive genes involved in ABA signaling and cold response regulatory networks were selected for quantification by qRT-PCR. This gene panel included the ABA signaling marker gene *AtKIN1* (AT3G63480) and five core components of the ICE1-CBF-COR cold response cascade: *AtCBF1* (AT4G25490), *AtCBF2* (AT4G25470), *AtCBF3* (AT4G25480), *AtCOR15a* (AT2G42540), and *AtCOR47* (AT1G20440) (Figure 9).

Under normal growth conditions, all detected genes exhibited comparably low basal expression levels across wild-type (WT), empty vector control (UL), and *AaMYB4*-overexpressing lines (S1, S3, S5), with no significant transcriptional differences observed across groups. In contrast, cold and drought stresses strongly induced the expression of these downstream genes across all genotypes. Notably, transgenic lines with constitutive *AaMYB4* expression showed significantly higher transcript levels of all six marker genes than WT and UL controls under stress conditions (Figure 9A–F).

## 3. Discussion

The MYB superfamily is one of the largest transcription factor families in higher plants, with members regulating diverse processes including cell cycle progression, primary and secondary metabolite biosynthesis, and signaling cascades underlying plant stress adaptation [41,42,43]. Accumulating evidence from transgenic functional validation studies demonstrates that, relative to wild-type (WT) plants, lines with elevated MYB transcriptional activity generally exhibit improved abiotic stress tolerance, which translates to markedly higher plant survival rates under adverse growth conditions such as low temperature, drought, and salinity [44,45].

Among all MYB subfamilies, R2R3-MYB proteins emerge as master regulators that mediate plant adaptive responses to a broad range of abiotic stresses. Functional studies in the model plant *Arabidopsis thaliana* have defined distinct stress-specific functions for multiple MYB homologs. Genes such as *AtMYB2*, *AtMYB44*, *AtMYB74*, and *AtMYB102* act as critical signal transducers under water-deficit conditions and enhance whole-plant drought tolerance via downstream transcriptional reprogramming [30,46]. By contrast, low-temperature signals strongly upregulate the expression of *AtMYB8*, *AtMYB14*, and *AtMYB15*, which function in the intricate molecular cascade underlying cold acclimation [47,48]. These stress-regulatory roles of R2R3-MYB transcription factors have also been extensively characterized in woody perennial fruit crops, with both positive and negative regulatory modes reported across species. In apple (*Malus domestica*), the R2R3-MYB transcription factor *MdMYB23* functions in cold tolerance regulation and proanthocyanidin biosynthesis [49]. Conversely, the pear R2R3-MYB transcription factor *MYB20* negatively regulates tolerance to both salt and cold stress [50]. In the context of postharvest cold adaptation, loquat *EjMYB15* confers cold tolerance to harvested fruit by transcriptionally upregulating antioxidant enzyme genes [51]. However, MYB-governed abiotic stress regulatory networks in the kiwiberry cultivar ‘Fenglü’ (*Actinidia arguta*) remain poorly characterized at a systematic level.

In this study, we used the kiwiberry cultivar ‘Fenglü’ as experimental material and successfully isolated the full-length coding sequence of *AaMYB4*. Bioinformatic analysis revealed that the full coding region of *AaMYB4* spans 726 base pairs and encodes a protein of 241 amino acid residues. In silico conserved domain analysis identified two tandem conserved SANT motifs within the deduced AaMYB4 protein sequence (Figure 1A), each harboring the conserved amino acid signatures characteristic of the MYB DNA-binding domain.

Pairwise sequence alignment of orthologous MYB proteins from diverse plant taxa and phylogenetic reconstruction revealed high sequence conservation between AaMYB4 and MYB homologs across a broad range of plant lineages. Phylogenetic tree analysis further demonstrated that AaMYB4 exhibits the highest sequence similarity to AcMYB4, an R2R3-MYB ortholog from *Actinidia chinensis* (Figure 1B). Three-dimensional homology modeling further confirmed that AaMYB4 possesses the characteristic conserved folds of R2R3-MYB family members (Figure 2B), and its overall tertiary structure shows marked similarity to other previously reported plant MYB transcription factors (Figure 2C).

These structural findings are highly consistent with prior reports of AcMYB4 from *Actinidia chinensis* cultivars, underscoring the strong evolutionary conservation of MYB transcription factors across the genus *Actinidia* and the wider plant kingdom. Conservation of these functional domains is hypothesized to underlie the conserved biological functions of MYB proteins across plant species. Collectively, these structural features support the conserved function of *AaMYB4* as a canonical R2R3-MYB factor and provide a basis for investigating its role in abiotic stress tolerance.

Transcription factors exert their transcriptional activation and repression functions primarily in the nucleus, and their subcellular localization provides a key basis for inferring protein function [52]. Consistent with most plant MYB factors, nuclear localization enables *AaMYB4* to bind cis-regulatory elements in target promoters and regulate downstream physiological and stress-responsive pathways. This nuclear localization pattern has been corroborated by studies across multiple plant species. For example, five MYB transcription factors in *Solanum lycopersicum* (*SlMYB306-like*, *SlMYB1L*, *SlMYB108*, *SlMYB72*, and *SlMYB75*) exhibit exclusive nuclear localization in transient expression assays. To determine the subcellular localization of *AaMYB4*, we constructed the recombinant fusion vector pCAMBIA1300-*AaMYB4*-GFP and delivered it into tobacco leaf epidermal cells via Agrobacterium-mediated transient transformation. Confocal laser scanning microscopy confirmed the exclusive nuclear localization of the *AaMYB4* fusion protein (Figure 3), which is consistent with the canonical subcellular localization pattern of functional transcription factors.

MYB family genes show highly divergent tissue-specific expression profiles across diverse plant taxa, reflecting their tissue-specific biological functions. In *Actinidia chinensis* kiwifruit, *AcMYB4* maintains high transcript abundance in young leaves and stems, but low expression in fruits and roots [53]. By contrast, *AcMYB113* exhibits ripening-specific upregulation in fruit flesh, with barely detectable transcript levels in leaves and stems [54]. Overall, most kiwiberry MYB genes exhibit constitutive expression across diverse vegetative and reproductive tissues, albeit at variable expression levels.

In this study, we quantified the transcript abundance of the target gene in five distinct tissues from ‘Fenglü’ kiwiberry—namely roots, stems, young leaves, mature leaves, and fruits (Figure 4). We performed qRT-PCR to systematically characterize the tissue-specific expression profile of *AaMYB4*, as well as its dynamic transcriptional changes in response to four treatments: cold, drought, salt stress, and exogenous abscisic acid (ABA). These parallel analyses elucidate the intrinsic association between the spatiotemporal expression pattern of *AaMYB4* and its functional role in mediating environmental adaptation.

Tissue-specific expression analysis revealed that *AaMYB4* is highly expressed in young leaves and stems of kiwiberry (Figure 4A). In response to abiotic stress stimuli, *AaMYB4* transcription was strongly induced and followed a unimodal kinetic pattern across the 24 h treatment period, corroborating its active role in plant adaptive responses to multiple adverse environmental cues. *AaMYB4* exhibited markedly stronger transcriptional responsiveness to cold and drought stresses: transcript levels peaked at 3 h after cold treatment, whereas maximum transcript abundance was detected at 6 h under drought conditions (Figure 4B,C).

Taken together, our transcript profiling data lead us to propose that *AaMYB4* most likely participates in the regulatory network governing cold and drought tolerance in kiwiberry. This expression pattern aligns with the well-established characteristics of most functionally characterized stress-responsive MYB transcription factors.

Three complementary experimental approaches—transient overexpression, VIGS, and stable heterologous transformation—were integrated in this study to systematically dissect the biological functions and underlying molecular mechanisms by which AaMYB4 mediates adaptive responses to cold and drought in kiwiberry.

Uncontrolled accumulation of ROS causes severe oxidative damage to intracellular macromolecules such as structural proteins. Both cold and drought stresses trigger oxidative stress cascades in plant cells and induce excessive ROS accumulation, including H_2_O_2_ and O_2_^−^ [55]. To mitigate oxidative damage, plant cells have evolved a multifaceted antioxidant defense system comprising low-molecular-weight protective metabolites and antioxidant enzymes, which collectively maintain ROS concentrations within physiologically tolerable ranges. This defense system serves as a core protective mechanism that alleviates ROS-induced cellular damage and preserves intracellular redox homeostasis [56]. MDA, a toxic byproduct of membrane lipid peroxidation triggered by ROS bursts, is cytotoxic to plant cells. Accordingly, MDA content is widely used as a reliable biochemical proxy for quantifying the severity of stress-induced cellular damage and assessing overall plant stress tolerance [57].

Exposure to abiotic stress disrupts the structural integrity and normal physiological function of plasma membranes, increasing membrane permeability and triggering massive electrolyte efflux [58]. Relative electrolyte leakage (EL) is therefore a well-established biochemical marker for quantifying the extent of membrane damage caused by adverse environmental conditions [59]. Under cold stress, plants synthesize osmoprotectants such as soluble sugars and proline to maintain osmotic balance, scavenge excess ROS, and alleviate stress-induced tissue damage. As a ubiquitous osmoprotectant, proline exhibits high water solubility and strong hydration capacity. It binds large amounts of free water molecules to preserve native protein folding, stabilize intracellular osmotic homeostasis, scavenge toxic hydroxyl radicals, and thus enhance plant stress tolerance [60].

In transient transformation assays using kiwiberry leaves, samples transiently overexpressing *AaMYB4* exhibited markedly reduced MDA contents, higher proline contents, and enhanced activities of three key antioxidant enzymes (SOD, POD, and CAT) compared with empty vector controls after cold treatment. Histochemical staining with DAB and NBT further revealed markedly reduced accumulation of H_2_O_2_ and O_2_^−^ in *AaMYB4*-overexpressing leaves, indicating enhanced cold tolerance (Figure 5).

Conversely, leaf samples with VIGS-mediated silencing of endogenous *AaMYB4* exhibited the opposite physiological phenotypes: higher MDA contents, lower proline contents, reduced antioxidant enzyme activities, excessive ROS accumulation, and severely impaired cold tolerance after cold treatment (Figure 6). Collectively, these results confirm that transient overexpression of *AaMYB4* significantly enhances cold tolerance in kiwiberry leaves, whereas targeted gene silencing diminishes this protective effect. These findings establish *AaMYB4* as a positive regulator of the cold acclimation response in *Actinidia arguta*.

To further validate the stress-mitigating function of *AaMYB4*, we introduced its full-length coding sequence into *Arabidopsis thaliana* via stable heterologous transformation and generated homozygous overexpression lines. Upon exposure to cold and drought treatments, transgenic overexpression seedlings showed significantly higher survival rates and less severe leaf necrotic lesions compared with wild-type (WT) and empty vector (UL) control lines.

Consistent with the results from transient assays in kiwiberry, stable heterologous expression of *AaMYB4* in *Arabidopsis thaliana* conferred enhanced tolerance to both cold and drought stresses. Under normal growth conditions, no visible morphological differences were observed among wild-type (WT), empty vector (UL) control, and *AaMYB4*-overexpressing lines (S1, S3, S5), indicating that *AaMYB4* overexpression imposes no obvious growth penalty under non-stress conditions (Figure 7A,B). After 12 h of cold stress or 10 days of drought treatment, WT and UL plants developed severe leaf wilting, chlorosis, and necrosis, whereas *AaMYB4*-overexpressing lines retained relatively intact leaf morphology. Following a 7-day recovery period, the survival rates of the three overexpression lines were 3.4–4.1-fold higher than those of the control groups under both stress conditions (Figure 7C).

In situ histochemical staining further revealed differential ROS accumulation across genotypes under stress conditions. Under control conditions, all lines exhibited negligible DAB and NBT signals, reflecting uniformly low basal ROS levels. Upon cold and drought treatments, WT and UL leaves showed strong brown (DAB) and blue (NBT) staining. In contrast, *AaMYB4*-overexpressing lines exhibited markedly weaker staining, indicating significantly reduced ROS accumulation in leaf tissues (Figure 7D). These results demonstrate that *AaMYB4* overexpression effectively alleviates abiotic stress-induced ROS overaccumulation.

To further elucidate the physiological basis underlying the enhanced stress tolerance conferred by *AaMYB4*, we quantified a panel of physiological and biochemical parameters in wild-type (WT), empty vector control, and *AaMYB4*-overexpressing *Arabidopsis* lines under control, cold, and drought conditions (Figure 8). Under non-stress conditions, all genotypes showed comparable levels of proline, chlorophyll, MDA, relative electrolyte leakage, ROS, and antioxidant enzyme activities, indicating that *AaMYB4* overexpression does not perturb basal physiological homeostasis.

Upon exposure to cold and drought stress, all lines exhibited typical stress-induced physiological changes, including proline accumulation, chlorophyll degradation, increased MDA content and relative electrolyte leakage, ROS burst, and enhanced antioxidant enzyme activities. However, the magnitude of these responses differed significantly between transgenic and control lines. *AaMYB4*-overexpressing lines accumulated significantly more proline than wild-type (WT) and UL controls under both stresses, suggesting that *AaMYB4* promotes osmotic adjustment to maintain cellular water homeostasis and preserve protein structural integrity under stress. Consistently, transgenic lines retained higher chlorophyll contents after stress treatment, indicating reduced damage to the photosynthetic apparatus.

As direct markers of oxidative membrane damage, MDA content and relative electrolyte leakage were markedly lower in *AaMYB4*-overexpressing lines than in controls under stress conditions, consistent with the reduced leaf necrosis and improved growth performance observed in the phenotypic assays. Consistent with the histochemical staining results, quantitative measurements confirmed that cold- and drought-induced H_2_O_2_ and O_2_^−^ accumulation was significantly attenuated in transgenic lines. This reduced ROS accumulation was accompanied by significantly higher activities of three core antioxidant enzymes—SOD, POD, and CAT—in *AaMYB4*-overexpressing lines.

Collectively, these findings suggest that enhanced antioxidant defense represents one key physiological mechanism by which *AaMYB4* alleviates oxidative damage under abiotic stress. The increased activities of antioxidant enzymes likely enhance cellular ROS scavenging capacity, thereby preserving intracellular redox homeostasis and attenuating lipid peroxidation. Beyond the antioxidant defense system, multiple complementary mechanisms likely act in concert with ROS scavenging to promote stress adaptation. For example, greater chlorophyll retention helps maintain photosynthetic capacity under adverse growth conditions.

MYB transcription factors function as central regulatory hubs in the complex signal transduction networks governing plant responses to diverse abiotic stresses. Their core regulatory function is to bind specific cis-regulatory elements in the promoters of target genes, thereby remodeling downstream transcriptional output and reprogramming a suite of physiological processes that collectively determine plant stress tolerance. Higher plants harbor multiple interconnected signaling cascades underlying stress acclimation, among which the ICE1-CBF-COR regulatory module and the ABA-dependent signal transduction pathway constitute the two core pathways orchestrating cold and drought tolerance.

Higher plants have evolved sophisticated multilayered regulatory systems to mitigate cellular damage caused by adverse growth environments, a conserved adaptive strategy mediated by the coordinated activation of multiple defense-related signaling cascades [61]. CBF transcription factors act as master regulatory hubs in the canonical ICE1-CBF-COR cold response pathway, which mediates plant adaptation to abiotic stress. Conserved CBF-targeted cis-regulatory elements have been identified across a wide range of plant species, and functional studies across diverse taxa confirm that CBF family genes are strongly transcriptionally induced in response to cold, drought, and other abiotic stress signals [62]. As core upstream regulators of cold acclimation, CBF proteins bind CRT elements in the COR47 promoter to activate its transcription and enhance plant freezing tolerance [63]. Drought-induced endogenous ABA accumulation further promotes the physical interaction between CBF proteins and CRT/DRE cis-elements, thereby driving robust expression of downstream stress-mitigating functional genes [64].

The peanut transcription factor *AhMYB30* exemplifies this dual regulatory mode: it confers freezing and salt stress tolerance in transgenic *Arabidopsis* plants via both the DREB/CBF and ABA signaling pathways [65]. Ectopic expression of DREB (Drought Response Element-Binding Protein) drives the transcriptional activation of multiple downstream stress marker genes, enhancing plant stress survival under multiple adverse conditions including drought, high salinity, and chilling stress [66]. This dual regulatory strategy integrating the CBF cascade and ABA signaling is not limited to herbaceous plants; it is also broadly conserved in perennial woody fruit crops. In Amur grape (*Vitis amurensis*), *VaMYB4a* mediates cold tolerance by coordinately modulating the CBF-COR cascade and ABA pathway, further corroborating that MYB-mediated coordination of CBF and ABA signaling represents a conserved mechanism across perennial woody species [67].

To investigate the molecular basis of *AaMYB4*-mediated stress tolerance, we quantified the transcript levels of six canonical stress-responsive marker genes involved in ABA signaling and the ICE1-CBF-COR cold signaling cascade (Figure 9). Under control conditions, all tested genes exhibited uniformly low basal expression levels across wild-type (WT), empty vector (UL) control, and *AaMYB4*-overexpressing lines, indicating that *AaMYB4* alone is not capable of inducing constitutive expression of these stress-responsive genes in the absence of stress.

Upon cold and drought stress, the expression of all six marker genes was strongly induced in all lines. Notably, *AaMYB4*-overexpressing lines exhibited significantly higher transcript levels of these genes than WT and UL controls under both stress conditions. *AtKIN1*, a well-characterized marker gene for the ABA-dependent stress signaling pathway, was consistently more strongly upregulated in transgenic lines under stress, suggesting that *AaMYB4* is positively correlated with ABA-mediated stress responses. In parallel, the core components of the ICE1-CBF-COR cascade—*AtCBF1*, *AtCBF2*, *AtCBF3*, *AtCOR15a*, and *AtCOR47*—all exhibited significantly higher expression levels in *AaMYB4*-overexpressing lines under cold and drought stresses, suggesting that *AaMYB4* may be involved in the CBF-mediated cold tolerance pathway.

These expression profiles demonstrate that the stress-tolerant phenotype of *AaMYB4*-overexpressing lines is accompanied by elevated transcript levels of genes in both the ABA-dependent and CBF-dependent stress signaling pathways. This dual involvement in two core stress signaling modules is consistent with previous reports on other stress-responsive MYB transcription factors, such as *AhMYB30* from peanut, which modulates both the DREB/CBF and ABA pathways to confer abiotic stress tolerance in transgenic *Arabidopsis*. Notably, our current data only support a correlative relationship between *AaMYB4* and the activation of these pathways. Whether *AaMYB4* directly regulates the transcription of these genes via promoter binding requires further validation using assays such as the yeast one-hybrid assay, electrophoretic mobility shift assay (EMSA), and chromatin immunoprecipitation (ChIP) assay.

**Figure 10:**
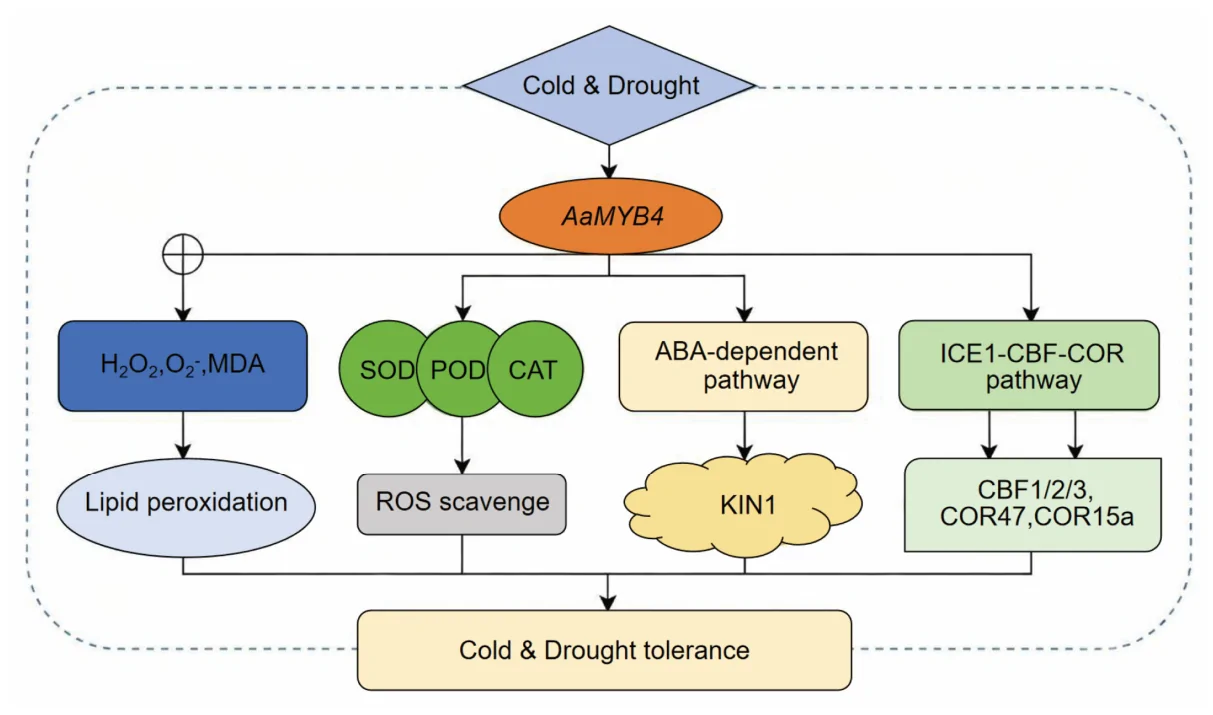
A possible model for the role of *AaMYB4* during plant tolerance to cold and drought stress.

Taken together, we conclude that *AaMYB4* participates in plant adaptive responses to cold and drought stress, and its function is positively correlated with the transcript levels of core genes in two signaling modules: the ABA-dependent signal transduction pathway and the ICE1-CBF-COR cold signaling cascade (Figure 10). These findings provide candidate gene resources for dissecting the abiotic stress regulatory network and advancing molecular breeding for stress tolerance in *Actinidia arguta*, and advance our understanding of MYB transcription factor functions in fruit crops. This study has two main limitations. First, the inferred regulatory mechanism is based only on physiological and expression data, with no direct evidence of *AaMYB4* target genes. Second, all functional assays were performed in heterologous *Arabidopsis*. Future work will screen direct targets of *AaMYB4* and validate its function in kiwiberry.

## 4. Materials and Methods

### 4.1. Plant Materials and Growth Conditions

The perennial kiwiberry cultivar *Actinidia arguta* ‘Fenglü’ was grown at the Horticultural Experimental Station of Northeast Agricultural University. Sterile tissue-culture plantlets were obtained using young stem segments as explants under aseptic conditions. Seeds of *Arabidopsis thaliana* ecotype Columbia and *Nicotiana benthamiana* were maintained in our laboratory. All plant materials were cultivated in a controlled growth chamber under uniform conditions: a temperature of 25 ± 1 °C, a light intensity of 3000 lux, a 16 h light/8 h dark photoperiod, and a relative humidity of 70–80% [68].

### 4.2. Experimental Methods

#### 4.2.1. Total RNA Extraction and cDNA Synthesis

Total RNA was extracted from young leaves of *A. arguta* ‘Fenglü’ using a Plant Total RNA Extraction Kit (Tiangen Biotech, Beijing, China) following the manufacturer’s protocol. RNA integrity and purity were assessed via 1% agarose gel electrophoresis and a NanoDrop 2000 UV spectrophotometer (Thermo Fisher Scientific, Waltham, MA, USA). First-strand cDNA was synthesized from high-quality total RNA using the PrimeScript™ RT Reagent Kit with gDNA Eraser (TaKaRa, Shiga, Japan) [69,70].

#### 4.2.2. Gene Cloning of AaMYB4

Based on previous cold stress transcriptome data from A. arguta, gene-specific primers for AaMYB4 were designed using Primer Premier 5.0 (designated AaMYB4-F/R; Table S1). The full-length coding sequence of AaMYB4 was amplified by reverse transcription PCR (RT-PCR) using cDNA from kiwiberry leaves as the template. The 25 μL PCR mixture consisted of 12.5 μL of 2× Taq PCR MasterMix, 1 μL of forward primer, 1 μL of reverse primer, 2 μL of cDNA template, and 8.5 μL of ddH_2_O. PCR amplification was performed under the following thermal conditions: initial denaturation at 94 °C for 3 min; 35 cycles of 94 °C for 30 s, 58 °C for 30 s, and 72 °C for 1 min; and a final extension at 72 °C for 10 min. The target PCR product was purified and ligated into the pMD19-T cloning vector (TaKaRa, Shiga, Japan). The resulting recombinant plasmids were transformed into Escherichia coli DH5α competent cells. Positive single colonies were selected and subjected to Sanger sequencing validation at Sangon Biotech (Shanghai, China) [71].

#### 4.2.3. Bioinformatic Analysis of AaMYB4

Protein physicochemical features were analyzed using ExPASy ProtParam. SMART and NCBI CDD detected conserved domains. MEGA 11 constructed a neighbor-joining phylogenetic tree (1000 bootstraps) based on GenBank homologous sequences. SOPMA and SWISS-MODEL separately predicted secondary and tertiary structures of *AaMYB4* [72,73].

#### 4.2.4. Subcellular Localization Assay

The pCAMBIA1300 vector harboring the 35S-GFP reporter cassette contains XbaI and KpnI restriction sites. We designed gene-specific primers with the corresponding restriction sites (*AaMYB4*-slF/slR; Table S2). The full-length coding sequence (CDS) of *AaMYB4*, excluding the stop codon, was amplified and inserted into the pCAMBIA1300-GFP backbone to generate the recombinant fusion expression vector *AaMYB4*-pCAMBIA1300. The empty pCAMBIA1300-GFP plasmid was used as the negative control.

Both the recombinant and empty vectors were transformed into *Agrobacterium tumefaciens* GV3101 competent cells. The prepared bacterial suspensions were infiltrated into the abaxial surfaces of *Nicotiana benthamiana* leaves. At 48–72 h post-infiltration, GFP fluorescence signals were observed and imaged using a laser scanning confocal microscope (Leica, Wetzlar, Germany) [74].

#### 4.2.5. Tissue-Specific and Stress-Induced Expression Patterns of AaMYB4

Tissue-specific expression analysis: Roots, stems, young leaves, mature leaves, and fruits of *A. arguta* ‘Fenglü’ were collected for total RNA extraction and subsequent cDNA synthesis. Stress-induced expression analysis: Seedlings were subjected to cold (4 °C), drought (20% PEG 6000), exogenous ABA (100 μmol/L), and high salinity (200 mmol/L NaCl) treatments. qRT-PCR was performed using the TB Green^®^ Premix Ex Taq™ II Kit on a StepOnePlus Real-Time PCR system (Thermo Fisher Scientific, Waltham, MA, USA). The housekeeping gene AaActin was used as the internal reference for transcript normalization. The relative transcript abundance of AaMYB4 was calculated using the 2^−ΔΔCt^ method [75].

#### 4.2.6. Transient Overexpression and VIGS Silencing of AaMYB4 in Kiwiberry Leaves

The full-length CDS of *AaMYB4* was cloned into the pCAMBIA1300 vector to construct the overexpression vector. The VIGS vector was constructed by inserting a 300 bp specific fragment of *AaMYB4* into the pTRV2 vector. The recombinant vectors were transformed into *A. tumefaciens* GV3101. The Agrobacterium solution was injected into the leaves of *A. arguta* tissue culture seedlings. After 48 h of culture, the leaves were treated with low temperature (4 °C) for 24 h. The physiological indexes (MDA, proline, SOD, POD, CAT) were measured, and ROS accumulation was detected by DAB and NBT staining [76].

#### 4.2.7. Acquisition of the Transgenic Plants

The construction of overexpression transgenic materials was carried out based on homologous recombination principles, and target overexpression plasmids were designed with Primer 5.0 software. The custom amplification primers for vector construction contained homologous overlapping sequences, restriction enzyme cleavage sites, and gene-specific amplification fragments (Table S2). We amplified the 5′ terminal fragment carrying the KpnI restriction site and the 3′ terminal fragment with the BamHI site of the *AaMYB4* full-length cDNA. These amplified fragments were ligated with linearized pCAMBIA1300 vector digested by corresponding restriction enzymes, and the recombinant *AaMYB4*-OE overexpression plasmid was generated via homologous recombination primer assembly and cloning techniques.

Recombinant overexpression plasmid and empty pCAMBIA1300 vector were stored at −80 °C. High-purity plasmids were extracted from activated bacterial cultures using a high-efficiency plasmid extraction kit (Cat. No. CW0500M). Sequencing-confirmed plasmids were transformed into *A. tumefaciens* GV3101 competent cells. Transgenic *Arabidopsis* plants were obtained via the classical floral dip transformation method [77,78].

The harvested T_1_ generation seeds were sterilized and sown on MS solid medium containing 50 mg/L kanamycin for primary positive seedling screening. Putative T_2_ transgenic lines were further verified by qRT-PCR. WT and UL plants were used as two independent negative control groups. Three lines (S1, S3, S5) exhibiting the highest transcript abundance of the target gene were selected from T_2_ generation materials and propagated to obtain homozygous T_3_ lines, which were employed as experimental materials for subsequent abiotic stress treatments and physiological measurements. The complete composition of culture media used for *Arabidopsis* genetic transformation is listed in Table S3.

#### 4.2.8. Cold and Drought Stress Treatments and Physiological Index Determination

WT, UL, and T_3_ homozygous *AaMYB4*-overexpressing *Arabidopsis* seedlings grown for approximately 40 days were used for stress treatments, with 20 uniform seedlings set for each group. For cold stress, seedlings were incubated at 4 °C for 12 h. For drought stress, water supply was suspended for 10 consecutive days, followed by a 7-day rewatering recovery period. Plant phenotypic changes were recorded before treatment, immediately after stress exposure, and after recovery. Multiple physiological and biochemical indices were systematically determined for all plant lines.

Proline content was measured using the acid ninhydrin colorimetric method [79]; total chlorophyll concentration was determined by organic solvent extraction [80]. The enzymatic activities of three antioxidant proteins were separately assayed: SOD via the NBT photoreduction method [81], peroxidase (POD) via the guaiacol chromogenic method [82], and CAT via UV spectrophotometry [82]. MDA accumulation was detected using the TBA color reaction [83]. H_2_O_2_ and O_2_^−^ contents were quantified via potassium iodide colorimetry [84] and hydroxylamine oxidation method [85], respectively. Relative electrolyte leakage was detected using a conductivity meter based on vacuum infiltration method [86].

#### 4.2.9. In Situ ROS Histochemical Staining

DAB and NBT staining were performed to visualize ROS accumulation in *Arabidopsis* leaves. For DAB staining, leaf samples from WT, UL, and transgenic lines were immersed in 1 mg/mL DAB solution and incubated at room temperature under dark conditions until brown staining appeared. Leaves were then decolorized with an ethanol–lactic acid–glycerol mixed solution (3:1:1) to remove chlorophyll for microscopic observation. For NBT staining, leaves were submerged in 0.5 mg/mL NBT solution until blue formazan precipitates formed, followed by complete chlorophyll removal using the decolorizing solution for photography and phenotypic analysis [87].

#### 4.2.10. Expression Analysis of Downstream Stress-Responsive Genes

Leaf samples of WT, UL, and transgenic *Arabidopsis* were collected before and after cold and drought stress treatments for total RNA extraction and cDNA synthesis. The transcriptional levels of stress-related genes from *Arabidopsis* were quantified by qRT-PCR using specific primer pairs listed in Table S1 [87].

#### 4.2.11. Statistical Analysis

All experiments in this study included at least three independent biological replicates. For quantitative assays including physiological index measurements and qRT-PCR, each biological sample was tested in three technical replicates. Histochemical staining assays were repeated independently three times, with no fewer than 5–6 leaves per genotype analyzed in each replicate. Prior to statistical analysis, the normality of data distribution and homogeneity of variance were tested for all datasets. One-way analysis of variance (ANOVA) followed by Duncan’s post hoc test was used for multi-group comparisons, and independent Student’s *t*-test was applied for pairwise comparisons between treatment and control groups. All quantitative data are presented as mean ± standard error (SE), and error bars in all figures uniformly represent standard error (SE). A *p*-value ≤ 0.05 was considered statistically significant, and *p* ≤ 0.01 was considered extremely significant. Data collation and statistical analysis were performed using Microsoft Excel 2019 and IBM SPSS Statistics 25.0, respectively, and graphs were generated using GraphPad Prism 8 [88].

## Data Availability

The original contributions presented in this study are included in the article/Supplementary Materials. Further inquiries can be directed to the corresponding authors.

## Supplementary Materials

The following supporting information can be downloaded at: https://www.mdpi.com/article/10.3390/plants15162426/s1, Figure S1: Electrophoretic bands of RNA extracted from samples; Figure S2: Amplified band of *AaMYB4* with a full-length size of 726 bp; Figure S3: Nucleotide and deduced amino acid sequences of *AaMYB4*; Figure S4: Instantaneous overexpression and silencing of *AaMYB4*; Figure S5: Screening of *AaMYB4* transgenic *Arabidopsis thaliana*; Table S1: The primer sequence of *AaMYB4*; Table S2: Sequence of primers for subcellular localization and genetic transformation of *AaMYB4*; Table S3: Composition of culture medium.

## References

[1] Das S., Shil S., Rime J., Alice A., Yumkhaibam T., Mounika V., Singh A., Kundu M., Lalhmangaihzuali H., Hazarika T. (2025). Phytohormonal signaling in plant resilience: Advances and strategies for enhancing abiotic stress tolerance. Plant Growth Regul..

[2] Gautam M., Kariyat R. (2026). Drought and Herbivory Have Selective Transgenerational Effects on Soybean Eco-Physiology, Defence and Fitness. Plant Cell Environ..

[3] Jing Z., Liu N., Zhang Z., Hou X. (2024). Research Progress on Plant Responses to Stress Combinations in the Context of Climate Change. Plants.

[4] Shi Y., Zhang Z., Yan Z., Chu H., Luo C. (2025). Tomato mitogen-activated protein kinase: Mechanisms of adaptation in response to biotic and abiotic stresses. Front. Plant Sci..

[5] Wu Y., Liu J., Zhao L., Wu H., Zhu Y., Ahmad I., Zhou G. (2026). Abiotic stress responses in crop plants: A multi-scale approach. J. Integr. Agric..

[6] Di Y., Lou S., Wang Z., Wang T., Niu X., Ji Z., Liu W., Chen S., Guo Z., Zheng S. (2026). The transcription factor *MYB52* regulates salt stress tolerance in tomato by modulating ion homeostasis and proline biosynthesis. Plant Physiol..

[7] Wang W., Kou J., Luo L., Wang Y., Zhang L., Yang Y., Yang N. (2026). Transcription factor *MYB102* regulates plant growth and tolerance to multiple abiotic stresses in *Arabidopsis thaliana*. Planta.

[8] Yong Y., Bi H., Li M., Zhu Y., Zhou Q., Xing W., Zheng S., Zhang L., Lyu Y., Song R. (2026). *LlR3MYB*-mediated flavonoid biosynthesis confers cold stress tolerance in *Lilium lancifolium* through the *LlDREB-LlCHS2* regulatory cascade. Hortic. Res..

[9] Wang Y.-X., Hu Y., Chen B.-F., Zhu Y.-F., Dawuda M.M., Svetla S. (2018). Physiological mechanisms of resistance to cold stress associated with 10 elite apple rootstocks. J. Integr. Agric..

[10] Li W., Li H., Wei Y., Han J., Wang Y., Li X., Zhang L., Han D. (2024). Overexpression of a *Fragaria vesca* NAM, ATAF, and CUC (NAC) Transcription Factor Gene (*FvNAC29*) Increases Salt and Cold Tolerance in *Arabidopsis thaliana*. Int. J. Mol. Sci..

[11] Yadav S.K. (2010). Cold stress tolerance mechanisms in plants. A review. Agron. Sustain. Dev..

[12] Cao J., Bao J., Lan S., Qin X., Ma S., Li S. (2024). Research progress on low-temperature stress response mechanisms and mitigation strategies in plants. Plant Growth Regul..

[13] Fan Y., Wang K., Wang M., Wang X., Li H., Wu Y. (2023). Production, functions and scavenging mechanisms of reactive oxygen species in plants under low-temperature stress. Maejo Int. J. Sci. Technol..

[14] Guan Y., Hwarari D., Korboe H., Ahmad B., Cao Y., Movahedi A., Yang L. (2023). Low temperature stress-induced perception and molecular signaling pathways in plants. Environ. Exp. Bot..

[15] Murtic S. (2026). Insights into Morpho-Physiological and Biochemical Responses of Plants to Heat Stress: An Overview. Russ. J. Plant Physiol..

[16] Sun S., Geng H., Sina Q., Fu H., Wu Y., Yang R., Kong W., Wang Y., Huang B. (2026). The transcription factor *StC3H14* enhances cold tolerance through the CBF-dependent pathway in potato. Plant Physiol..

[17] Wang G., Xu X., Lu H., Mi M., Qv T., Shen Y., Ran J., Yang D., Li Y., Liu T. (2025). The bZIP transcription factor *BcbZIP72* positively regulates cold tolerance through CBF-dependent pathways in non-heading Chinese cabbage. Plant Physiol. Biochem..

[18] Liu S., Xing M., Yin X. (2026). MAPK regulates secondary metabolism and abiotic stress in horticultural and medicinal plants. Hortic. Res..

[19] Qu X., Zou J., Wang J., Yang K., Wang X., Le J. (2022). A Rice R2R3-Type MYB Transcription Factor *OsFLP* Positively Regulates Drought Stress Response via OsNAC. Int. J. Mol. Sci..

[20] Blanco E., Curci P.L., Manconi A., Sarli A., Zuluaga D.L., Sonnante G. (2022). R2R3-MYBs in Durum Wheat: Genome-Wide Identification, Poaceae-Specific Clusters, Expression, and Regulatory Dynamics Under Abiotic Stresses. Front. Plant Sci..

[21] Shinozaki K., Yamaguchi-Shinozaki K. (2000). Molecular responses to dehydration and low temperature: Differences and cross-talk between two stress signaling pathways. Curr. Opin. Plant Biol..

[22] Zhao H., Liu W., Hu Y., Liu X., He Y. (2008). Cis-regulatory element-based genome-wide identification of DREB1/CBF targets in *Arabidopsis*. Prog. Nat. Sci.-Mater. Int..

[23] Li J., Han G., Sun C., Sui N. (2019). Research advances of MYB transcription factors in plant stress resistance and breeding. Plant Signal. Behav..

[24] Qian Y., Yan J., Luo C., Li Y., Wu Y., Liu W., Liu W., Xie D., Jiang B. (2025). Genome-wide analysis of the MYB gene family and functional analysis of *BhMYB79* in wax gourd. Hortic. Plant J..

[25] Hwang J., Kim S., Son H., Kwak J. (2025). MYB Transcription Factors in Plant Developmental Plasticity. J. Plant Biol..

[26] Imran M., Wu Q., Chen G., Zhou L. (2025). Multifaceted roles and regulatory mechanisms of MYB transcription factors in plant development, secondary metabolism, and stress adaptation: Current insights and future prospects. GM Crops Food-Biotechnol. Agric. Food Chain.

[27] Jiang C., Gu X., Peterson T. (2004). Identification of conserved gene structures and carboxy-terminal motifs in the Myb gene family of *Arabidopsis* and *Oryza sativa* L. ssp indica. Genome Biol..

[28] Sun J., Xu J., Qu W., Han X., Qiu C., Gai Z., Zhai J., Qin R., Liu H., Wu Z. (2023). Genome-wide analysis of R2R3-MYB transcription factors reveals their differential responses to drought stress and ABA treatment in desert poplar (*Populus euphratica*). Gene.

[29] Yang J., Li N., Li M., Yi R., Qiu L., Wang K., Zhao S., Ma F., Mao K. (2025). The *MdHB7L-MdICE1L-MdHOS1* Module Fine-Tunes Apple Cold Response via CBF-Dependent and CBF-Independent Pathways. Adv. Sci..

[30] Cominelli E., Galbiati M., Vavasseur A., Conti L., Sala T., Vuylsteke M., Leonhardt N., Dellaporta S.L., Tonelli C. (2005). A Guard-Cell-Specific MYB Transcription Factor Regulates Stomatal Movements and Plant Drought Tolerance. Curr. Biol..

[31] Seo P.J., Xiang F., Qiao M., Park J.-Y., Lee Y.N., Kim S.-G., Lee Y.-H., Park W.J., Park C.-M. (2009). The *MYB96* Transcription Factor Mediates Abscisic Acid Signaling during Drought Stress Response in *Arabidopsis*. Plant Physiol..

[32] Gao S., Zhang Y.L., Yang L., Song J.B., Yang Z.M. (2014). *AtMYB20* is negatively involved in plant adaptive response to drought stress. Plant Soil.

[33] Ren C., Luo G., Li X., Yao A., Liu W., Zhang L., Wang Y., Li W., Han D. (2023). *MxFRO4* confers iron and salt tolerance through up-regulating antioxidant capacity associated with the ROS scavenging. J. Plant Physiol..

[34] Liu R., Shen Y., Wang M., Liu R., Cui Z., Li P., Wu Q., Shen Q., Chen J., Zhang S. (2023). *GhMYB102* promotes drought resistance by regulating drought-responsive genes and ABA biosynthesis in cotton (*Gossypium hirsutum* L.). Plant Sci..

[35] Peng J., Liu E., Wu Y., Wang J., Xu M., Li N., Hao X., Ding C., Wang X., Wang L. (2026). *CsWRKY57L* enhances freezing tolerance through flavonoid accumulation and modulates growth via SWEETs in tea plants. Hortic. Plant J..

[36] Li Y., Song Z., Zhan X., Li X., Ye L., Lin M., Wang R., Huang H., Guo J., Sun L. (2025). Chromosome-level genome assembly assisting for dissecting mechanism of anthocyanin regulation in kiwifruit (*Actinidia arguta*). Mol. Hortic..

[37] Song Z., Li Y., Zhan X., Li X., Ye L., Lin M., Wang R., Sun L., Chen J., Fang J. (2025). *AaMYB61-like* and *AabHLH137* jointly regulate anthocyanin biosynthesis in *Actinidia arguta*. BMC Plant Biol..

[38] Wang T., Sui Y., Du X., Zhang S., Chen L. (2025). A comprehensive review of post-harvest ripening, preservation and processing for *Actinidia arguta* (mini kiwi). J. Stored Prod. Res..

[39] Ren W., Cao L., Liu L., Xiang M., Zhang X., Sun J., Tang N., Wang L., Zhang C., Liu Y. (2025). The transcription factor *MYB78* negatively regulates cold stress tolerance in *Actinidia chinensis*. Plant Physiol..

[40] Yan M., Li X., Ji X., Gang B., Li Y., Li Z., Wang Y., Guo H. (2025). An R2R3-MYB transcription factor *PdbMYB6* enhances drought tolerance by mediating reactive oxygen species scavenging, osmotic balance, and stomatal opening. Plant Physiol. Biochem..

[41] Bhatt P., Gurav T., Kondhare K., Giri A. (2025). MYB proteins: Versatile regulators of plant development, stress responses, and secondary metabolite biosynthetic pathways. Int. J. Biol. Macromol..

[42] Wu X., Xia M., Su P., Zhang Y., Tu L., Zhao H., Gao W., Huang L., Hu Y. (2024). MYB transcription factors in plants: A comprehensive review of their discovery, structure, classification, functional diversity and regulatory mechanism. Int. J. Biol. Macromol..

[43] Zhang D., Zhou H., Zhang Y., Zhao Y., Zhang Y., Feng X., Lin H. (2025). Diverse roles of MYB transcription factors in plants. J. Integr. Plant Biol..

[44] Biswas D., Gain H., Mandal A. (2023). MYB transcription factor: A new weapon for biotic stress tolerance in plants. Plant Stress.

[45] Yu Y., Zhang S., Yu Y., Cui N., Yu G., Zhao H., Meng X., Fan H. (2023). The pivotal role of MYB transcription factors in plant disease resistance. Planta.

[46] Hoeren F., Dolferus R., Wu Y., Peacock W., Dennis E. (1998). Evidence for a role for *AtMYB2* in the induction of the *Arabidopsis* alcohol dehydrogenase gene ADH1 by low oxygen. Genetics.

[47] Chen Y., Chen Z., Kang J., Kang D., Gu H., Qin G. (2013). *AtMYB14* Regulates Cold Tolerance in *Arabidopsis*. Plant Mol. Biol. Report..

[48] Zhang L., Zhao G., Xia C., Jia J., Liu X., Kong X. (2012). Overexpression of a wheat MYB transcription factor gene, *TaMYB56-B*, enhances tolerances to freezing and salt stresses in transgenic *Arabidopsis*. Gene.

[49] An J., Li R., Qu F., You C., Wang X., Hao Y. (2018). R2R3-MYB transcription factor *MdMYB23* is involved in the cold tolerance and proanthocyanidin accumulation in apple. Plant J..

[50] Liu Z., Qiao Z., Ren Y., An J., Xu S., Gu K., Zheng P., Chen S., Xue C., Wu J. (2025). MYB20, an R2R3-type MYB transcription factor, negatively regulates salt and cold stress tolerance in pears. Plant Physiol. Biochem..

[51] Liang W., Wan J., Lin J., Wu Y., Su W., Fan Z. (2026). *EjMYB15* Improves Cold Tolerance of Postharvest Loquat Fruit via Upregulating Antioxidant Enzyme Genes. Foods.

[52] An Y., Xiaoyan D., WenHao Z. (2012). A R2R3-type MYB gene, *OsMYB2*, is involved in salt, cold, and dehydration tolerance in rice. J. Exp. Bot..

[53] Liang D., Deng H., Deng Q., Lin L., Lv X., Wang J., Wang Z., Xiong B., Zhao X., Xia H. (2020). Dynamic Changes of Phenolic Compounds and Their Associated Gene Expression Profiles Occurring during Fruit Development and Ripening of the Donghong Kiwifruit. J. Agric. Food Chem..

[54] Brian L., Warren B., McAtee P., Rodrigues J., Nieuwenhuizen N., Pasha A., David K.M., Richardson A., Provart N.J., Allan A.C. (2021). A gene expression atlas for kiwifruit (*Actinidia chinensis*) and network analysis of transcription factors. BMC Plant Biol..

[55] Noctor G., Mhamdi A., Foyer C.H. (2014). The Roles of Reactive Oxygen Metabolism in Drought: Not So Cut and Dried. Plant Physiol..

[56] Wang Y., Branicky R., Noe A., Hekimi S. (2018). Superoxide dismutases: Dual roles in controlling ROS damage and regulating ROS signaling. J. Cell Biol..

[57] Xie Z., Zhang Z., Zou X., Huang J., Ruas P., Thompson D., Shen Q. (2005). Annotations and functional analyses of the rice WRKY gene superfamily reveal positive and negative regulators of abscisic acid signaling in aleurone cells. Plant Physiol..

[58] Dumanovic J., Nepovimova E., Natic M., Kuca K., Jacevic V. (2021). The Significance of Reactive Oxygen Species and Antioxidant Defense System in Plants: A Concise Overview. Front. Plant Sci..

[59] Chan Z., Wang Y., Cao M., Gong Y., Mu Z., Wang H., Hu Y., Deng X., He X.J., Zhu J.K. (2016). *RDM4* modulates cold stress resistance in *Arabidopsis* partially through the CBF-mediated pathway. New Phytol..

[60] Rubio M.C., Bustos-Sanmamed P., Clemente M.R., Becana M. (2009). Effects of salt stress on the expression of antioxidant genes and proteins in the model legume *Lotus japonicus*. New Phytol..

[61] Shinozaki K., Yamaguchi-Shinozaki K., Seki M. (2003). Regulatory network of gene expression in the drought and cold stress responses. Curr. Opin. Plant Biol..

[62] Carvallo M.A., Pino M.-T., Jeknic Z., Zou C., Doherty C.J., Shiu S.-H., Chen T.H.H., Thomashow M.F. (2011). A comparison of the low temperature transcriptomes and CBF regulons of three plant species that differ in freezing tolerance: *Solanum commersonii*, *Solanum tuberosum*, and *Arabidopsis thaliana*. J. Exp. Bot..

[63] Jaglo-Ottosen K., Gilmour S., Zarka D., Schabenberger O., Thomashow M. (1998). *Arabidopsis CBF1* overexpression induces COR genes and enhances freezing tolerance. Science.

[64] Yamaguchi-Shinozaki K., Shinozaki K. (2001). Improving plant drought, salt and freezing tolerance by gene transfer of a single stress-inducible transcription factor. Novartis Foundation Symposium 236—Rice Biotechnology: Improving Yield, Stress Tolerance and Grain Quality.

[65] Chen N., Pan L., Yang Z., Su M., Xu J., Jiang X., Yin X., Wang T., Wan F., Chi X. (2023). A MYB-related transcription factor from peanut, *AhMYB30*, improves freezing and salt stress tolerance in transgenic Arabidopsis through both DREB/CBF and ABA-signaling pathways. Front. Plant Sci..

[66] Ryu H., Cho Y.-G. (2015). Plant hormones in salt stress tolerance. J. Plant Biol..

[67] Yu Q., Sun Y., Xie Y., Li J., Wang R., Zheng Q., Liu C., Zhang N., Xu W. (2026). Amur grape *VaMYB4a* mediates grapevine cold tolerance via dual regulation of CBF-COR and ABA pathways. J. Integr. Agric..

[68] Han D.G., Shi Y., Wang B., Liu W., Yu Z.Y., Lv B.-Y., Yang G.H. (2015). Isolation and Preliminary Functional Analysis of *MxCS2*: A Gene Encoding a Citrate Synthase in *Malus xiaojinensis*. Plant Mol. Biol. Report..

[69] Han D.G., Yang G.H., Xu K.D., Shao Q., Yu Z.Y., Wang B., Ge Q.L., Yu Y. (2013). Overexpression of a *Malus xiaojinensis Nas1* Gene Influences Flower Development and Tolerance to Iron Stress in Transgenic Tobacco. Plant Mol. Biol. Report..

[70] Zhang L., Wang Y., Zhang X., Zhang M., Han D., Qiu C., Han Z. (2012). Dynamics of phytohormone and DNA methylation patterns changes during dormancy induction in strawberry (*Fragaria* × *ananassa* Duch.). Plant Cell Rep..

[71] Han D., Shi Y., Yu Z., Liu W., Lv B., Wang B., Yang G. (2015). Isolation and functional analysis of MdCS1: A gene encoding a citrate synthase in *Malus domestica* (L.) Borkh. Plant Growth Regul..

[72] Han D.G., Wang Y., Zhang L., Ma L., Zhang X.Z., Xu X.F., Han Z.H. (2012). Isolation and functional characterization of *MxCS1*: A gene encoding a citrate synthase in *Malus xiaojinensis*. Biol. Plant..

[73] Wang X., Li Y., Chen Z., Li L., Li Q., Geng Z., Liu W., Hou R., Zhang L., Han D. (2025). *MbWRKY50* confers cold and drought tolerance through upregulating antioxidant capacity associated with ROS scavenging. J. Plant Physiol..

[74] Han D., Wang L., Wang Y., Yang G., Gao C., Yu Z., Li T., Zhang X., Ma L., Xu X. (2013). Overexpression of *Malus xiaojinensis CS1* gene in tobacco affects plant development and increases iron stress tolerance. Sci. Hortic..

[75] Han D., Wang Y., Zhang Z., Pu Q., Ding H., Han J., Fan T., Bai X., Yang G. (2017). Isolation and functional analysis of *MxCS3*: A gene encoding a citrate synthase in *Malus xiaojinensis*, with functions in tolerance to iron stress and abnormal flower in transgenic *Arabidopsis thaliana*. Plant Growth Regul..

[76] Li Y., Tan Z., Liu Y., Peng Y., Liu C. (2025). Root-Specific Overexpression of the *CmDUF239-1* Gene Enhances Heat Tolerance in Melon Seedlings by Upregulating Antioxidant Enzymes Activities, Proline Content, and Expression of Heat Shock Protein-Related Genes. Horticulturae.

[77] Han D., Zhang Z., Ni B., Ding H., Liu W., Li W., Chai L., Yang G. (2018). Isolation and functional analysis of *MxNAS3* involved in enhanced iron stress tolerance and abnormal flower in transgenic Arabidopsis. J. Plant Interact..

[78] Ren C., Li Z., Song P., Wang Y., Liu W., Zhang L., Li X., Li W., Han D. (2023). Overexpression of a Grape MYB Transcription Factor Gene *VhMYB2* Increases Salinity and Drought Tolerance in *Arabidopsis thaliana*. Int. J. Mol. Sci..

[79] Li P., Zheng X., Liu Y., Zhu Y. (2014). Pre-storage application of oxalic acid alleviates chilling injury in mango fruit by modulating proline metabolism and energy status under chilling stress. Food Chem..

[80] Zhang L., Zhu L., Xu Y., Lü L., Li X., Li W., Liu W., Ma F., Li M., Han D. (2023). Genome-wide identification and function analysis of the sucrose phosphate synthase *MdSPS* gene family in apple. J. Integr. Agric..

[81] Zhang L., Xing L., Dai J., Li Z., Zhang A., Wang T., Liu W., Li X., Han D. (2024). Overexpression of a Grape WRKY Transcription Factor *VhWRKY44* Improves the Resistance to Cold and Salt of *Arabidopsis thaliana*. Int. J. Mol. Sci..

[82] Zhang L., Pei Y., Wang H., Jin Z., Liu Z., Qiao Z., Fang H., Zhang Y. (2015). Hydrogen Sulfide Alleviates Cadmium-Induced Cell Death through Restraining ROS Accumulation in Roots of *Brassica rapa* L. ssp pekinensis. Oxidative Med. Cell. Longev..

[83] Xu F., Liu S., Liu Y., Xu J., Liu T., Dong S. (2019). Effectiveness of lysozyme coatings and 1-MCP treatments on storage and preservation of kiwifruit. Food Chem..

[84] Li Y., Zhong J., Huang P., Shao B., Li W., Liu W., Wang Y., Xie L., Han M., Han D. (2022). Overexpression of *MxFRO6*, a FRO gene from *Malus xiaojinensis*, increases iron and salt tolerance in *Arabidopsis thaliana*. In Vitro Cell. Dev. Biol.-Plant.

[85] Espinosa F., Garrido I. (2022). Thallium induced increases on O2^−^, H_2_O_2_, NO and H_2_S production, and morpho-physiological alterations in *Ditrichia viscosa* plants. Free Radic. Biol. Med..

[86] Zhang L., Xu Y., Luo Z., Lyu L., Wang C., Zhu L., Ma F., Li M., Han D. (2025). Overexpression of a transcription factor *MdWRKY126* altered soluble sugar accumulation in apple and tomato fruit. Hortic. Plant J..

[87] Han D., Hou Y., Ding H., Zhou Z., Li H., Yang G. (2018). Isolation and Preliminary Functional Analysis of *MbWRKY4* Gene Involved in Salt Tolerance in Transgenic Tobacco. Int. J. Agric. Biol..

[88] Chen Z., Wang J., Li W., Chen X., Zhao C., Guo Y., Li Y., Chen Z., Li X., Han D. (2025). *Arabidopsis thaliana* Plants’ Overexpression of the MYB Transcription Factor *VhMYB60* in the Face of Stress Hazards Enhances Salt and Cold Tolerance. Int. J. Mol. Sci..

