# Supplementary material for: Actinidia arguta AaMYB4 Confers Cold and Drought Tolerance Through Up-Regulating Antioxidant Capacity Associated with the ROS Scavenging
Source: Plants (Basel). 2026 Aug 9;15(16):2426. doi: 10.3390/plants15162426 (PMC13516710; doi:10.3390/plants15162426)
Supplement: Supplementary file 1 [file plants-15-02426-s001.zip › Supplementary Table.pdf]

Table S1.The primer sequence of *AaMYB4*

Primer	Sequence of primer（5'-3'）
AaMYB4-F	ATGGGAAGGTCTCCATGTTGTG
AaMYB4-R	TTTCATCTCCAAGCTTCTATAATCC

Table S2. Sequence of primers for subcellular localization and genetic transformation of AaMYB4

Primer	Sequence of primer（5'-3'）
AaMYB4-slF	GACGTCTTCGAGCTCGGTACCATGGGAAGGTCTCCATGTTGTG
AaMYB4-slR	CATGTCGACTCTAGAGGATCCTTTCATCTCCAAGCTTCTATAATCC

Table S3. Composition of culture medium

culture medium（1 L）	MS	sucrose	agar	SilwetL-77	100 mg/ml Kana
Sowing culture medium	4.3 g	30 g	7.5 g	—	—
Bacterial suspension culture medium	4.3 g	50 g	—	300 μL	—
Screening Culture Medium	4.3 g	30 g	7.5 g	—	500 μL
